# A novel mechanism of cone photoreceptor adaptation

**DOI:** 10.1371/journal.pbio.2001210

**Published:** 2017-04-12

**Authors:** Marcus H. C. Howlett, Robert G. Smith, Maarten Kamermans

**Affiliations:** 1 Retinal Signal Processing lab, Netherlands Institute for Neuroscience, Amsterdam, The Netherlands; 2 Department of Neuroscience, University of Pennsylvania, Philadelphia, Pennsylvania, United States of America; 3 Department of Genome Analysis, Academic Medical Center, University of Amsterdam, The Netherlands; Yale University, United States of America

## Abstract

An animal’s ability to survive depends on its sensory systems being able to adapt to a wide range of environmental conditions, by maximizing the information extracted and reducing the noise transmitted. The visual system does this by adapting to luminance and contrast. While luminance adaptation can begin at the retinal photoreceptors, contrast adaptation has been shown to start at later stages in the retina. Photoreceptors adapt to changes in luminance over multiple time scales ranging from tens of milliseconds to minutes, with the adaptive changes arising from processes within the phototransduction cascade. Here we show a new form of adaptation in cones that is independent of the phototransduction process. Rather, it is mediated by voltage-gated ion channels in the cone membrane and acts by changing the frequency response of cones such that their responses speed up as the membrane potential modulation depth increases and slow down as the membrane potential modulation depth decreases. This mechanism is effectively activated by high-contrast stimuli dominated by low frequencies such as natural stimuli. However, the more generally used Gaussian white noise stimuli were not effective since they did not modulate the cone membrane potential to the same extent. This new adaptive process had a time constant of less than a second. A critical component of the underlying mechanism is the hyperpolarization-activated current, I_h_, as pharmacologically blocking it prevented the long- and mid- wavelength sensitive cone photoreceptors (L- and M-cones) from adapting. Consistent with this, short- wavelength sensitive cone photoreceptors (S-cones) did not show the adaptive response, and we found they also lacked a prominent I_h_. The adaptive filtering mechanism identified here improves the information flow by removing higher-frequency noise during lower signal-to-noise ratio conditions, as occurs when contrast levels are low. Although this new adaptive mechanism can be driven by contrast, it is not a contrast adaptation mechanism in its strictest sense, as will be argued in the Discussion.

## Author summary

An animal’s ability to survive depends on its ability to adapt to a wide range of light conditions, by maximizing the information flow through the retina. Here, we show a new form of adaptation in cone photoreceptors that helps them optimize the information they transmit by adjusting their response kinetics to better match the visual conditions. The adaptive mechanism we describe is independent of the cone phototransduction process and is instead mediated by membrane processes in which the hyperpolarization-activated current, I_h_, plays a critical role. Consistent with the critical role of this current, we also found that cones sensitive to short wavelengths lacked a prominent I_h_ current and did not show this new form of adaptation. As voltage-dependent processes underlie the adaptational mechanism, it is only apparent when the stimuli are able to sufficiently modulate the membrane potential of cones. This happens with natural stimuli, which are able to deliver high levels of “effective” contrast. However, even though this new adaptive mechanism can be driven by contrast, we argue in the Discussion that in its strictest sense it is not a contrast adaptation mechanism per se.

## Introduction

A natural environment is an ever-changing sensory landscape. Sensory systems adapt to these changes, increasing an animal’s ability to extract important information and survive under a wide range of conditions. In the natural world, the mean luminance and variations around the mean luminance—i.e., contrast—are poorly correlated [1]. This relative independence of contrast and luminance is reflected in the functional organization of the visual system, as retinal neurons adapt independently to these two basic features of natural scenes [1]. In vertebrates, most cone luminance adaptation takes place in the phototransduction cascade [2]. By adapting to the luminance level, photoreceptors primarily encode contrast.

Although contrast levels can vary widely between different natural scenes and even between locations within a natural scene, they have strong regularities. In natural scenes, the power spectrum declines as the frequency increases in a 1/f^β^ fashion (0.7 < β < 3) [3,4], reflecting the preponderance of larger and slower-moving objects over smaller and faster ones. Consequently, as noise in the responses of cones declines in power with frequency at a slower rate (~0.3 < β < 0.4) [5–8], the signal-to-noise ratio (SNR) of a cone’s response will decrease with increasing frequency. At some point, the signal becomes indistinguishable from the noise, at which point mostly noise is transmitted to the rest of the visual system. This “threshold frequency” will depend on the contrast level. In low-contrast conditions, it will occur at a lower frequency than in high-contrast conditions.

Cone noise largely originates from the outer segment and has intrinsic and extrinsic sources. One primary intrinsic source, the gating transition in cyclic guanosine monophosphate (cGMP)-gated channels, generates noise ranging from low frequencies to those well beyond the flicker fusion frequency. On the other hand, the photoreceptor inner segment conductances contribute little cone response noise [8]. In principle, the inner segment membrane acts as a band-pass filter, thereby reducing the amount of higher-frequency outer-segment noise transmitted by cones. However, to do this optimally, the filter should adapt such that its cutoff frequency remains close to the threshold frequency in all stimulus conditions. If this is not the case, information will be lost or additional noise will enter the system. Adaptive filtering by photoreceptors would maximize the amount of sensory information extracted from a natural scene and reduce the amount of noise transmitted under the various contrast conditions encountered. Thus, the entire visual system would benefit if photoreceptors could act as activity-dependent adaptive filters.

In this paper, we show that long- and mid-wavelength sensitive cones (L- and M-cones) do indeed act as activity-dependent adaptive filters. Cone frequency responses change such that L- and M-cones become slower under stimuli that induce minor membrane potential deflections as happens in low-contrast conditions. When the induced voltage deflections are larger such as in high-contrast conditions, L- and M-cones responses speed up. This form of adaptation has a time constant of less than a second, and the cone hyperpolarization-activated current (I_h_) is a critical component of the underlying mechanism. Consistent with this result is the finding that short-wavelength sensitive cones (S-cones) have a significantly smaller I_h_ compared to L- and M-cones and contrast did not affect their frequency responses.

## Results

### Cone responses to a naturalistic chromatic time series of intensities

**Fig 1:**
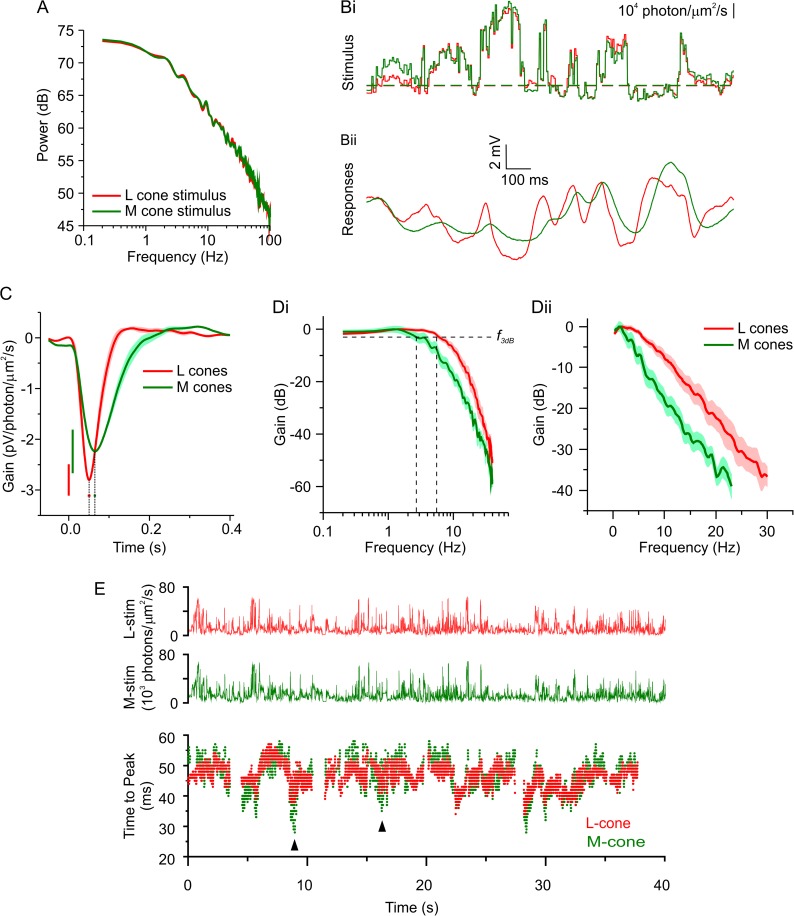
L- and M-cones adapt to contrast—natural time series of chromatic intensity (NTSCI). (A) Power spectral density of the long- (red) and mid- (green) wavelength sensitive cone photoreceptors (L- and M-cones) -specific NTSCI stimuli. (Bi) A small section of the two cone-specific versions of the NTSCI and the corresponding response (Bii) of a representative L- (red) and M- (green) cone. (C, D) The mean ± standard error of the mean (SEM) impulse-response functions (C) and normalized frequency response amplitude (D) of four L- and four M-cones under the NTSCI condition. C and D are both representations of the characteristics of the stimulus-response transfer function to the entire NTSCI (i.e., the full 40 s). In C, the SEMs of the peak amplitudes are indicated by the color-coded vertical bars, and the SEMs for the time that the maximal peak value occurs (time to peak, T2P) are indicated by the color-coded horizontal bars. Dii shows the data on a linear frequency axis to highlight the differences at higher frequencies, and Di shows the method for determining the frequency at which the frequency response amplitude had dropped by −3dB (*f*_*3dB*_). (E) Upper, the L- and M-cone-specific NTSCI stimuli. Lower, the T2P of impulse-response functions calculated over 1-s periods at random locations during the NTSCI for a representative L- and M-cone. Arrowheads indicate two areas where the M-cone T2P occurred sooner than for the L-cone. The data to generate this figure can be found in S1 Data.

To study the behavior of cones under naturalistic stimulus conditions, we recorded the voltage light responses of goldfish cones to a natural time series of chromatic intensities (NTSCI). Cones were stimulated with a 20-μm spot of light, which was modulated by a 40-s-long NTSCI segment obtained from van Hateren's natural-stimulus collection [9]. The NTSCI segment was preceded and followed by a 4.5-s period of white light with the same mean luminance. We first calculated the “cone-specific” stimuli the NTSCI presented to the L-or M-cones in photons/μm^2^/s absorbed by the specific cone types (see Fig 1) by using the measured spectral output of the stimulus-generating light-emitting diodes (LEDs) and the spectral sensitivity of goldfish cones [10]. Fig 1A shows the frequency distributions of both the L-and the M-cone-specific stimuli, which are approximately equal. The mean luminance was approximately the same (0.04 log unit difference), whereas the contrast experienced by the M-cones was about 15% lower than that for the L-cones.

The NTSCI was repeated multiple times for as long as the light response of the cones remained stable (4.8 ± 0.25 times; *n* = 8). Fig 1Bi shows a 1.5-s section of the two cone-specific NTSCI stimuli, and Fig 1Bii the corresponding responses for an L- (red traces) and an M- (green traces) cone. Although the L-and M-cone responses were rather similar, scrutinizing the traces revealed significant differences in their response kinetics. For example, in the section shown in Fig 1Bii, the M-cone response clearly lags behind the L-cone response and did not follow the rapidly changing aspects of the stimulus with the same fidelity. To investigate these differences further, we calculated the stimulus-response transfer functions for the L- and M- cones for the 40-s NTSCI period. When expressed as impulse-response functions (Fig 1C), the M-cone (*n* = 4) function peaked 14.3 ± 4.6 ms later (*p* = 0.021), and its full width at half maximum (FWHM) was 38.2 ± 8.06 ms (*p* = 0.0032) wider than for L-cones (*n* = 4). These differences indicate that the average M-cone response to the NTSCI was slower than that of the L-cones. These characteristics are also apparent when the transfer functions are expressed as frequency response curves (Fig 1Di; non-normalized data in S1A Fig). The larger lower-frequency content of the M-cone response is demonstrated by the higher gain levels at all frequencies lower than 2 Hz, compared to that of the L-cones (0.002 < *p* < 0.047 in all cases). Similarly, the faster response kinetics of the L-cones are demonstrated by the slower rate of decline of gain for higher frequencies (Fig 1Dii, −1.4 ± 0.18 dB/Hz) compared to the M-cones (−1.9 ± 0.06 dB/Hz; *p* = 0.032). The faster response kinetics of the L-cones are also indicated by their broader bandwidths, which is conventionally defined as the frequency at which the gain had reduced by 3dB (*f*_*3dB*_). The L-cone *f*_*3dB*_ (6.3 ± 1.02 Hz) was nearly twice that of M-cones (3.2 ± 0.37 Hz, *p* = 0.032). To obtain a better intuition about the size of the effect, we estimated the integration time by dividing the integral of the initial hyperpolarizing lobe of the impulse-response function by its maximum amplitude [11] and found that the integration time of the M-cones was 75% longer than for the L-cones (91.6 ± 7.03 ms versus 51.6 ± 5.46 ms; *p* = 0.0041).

We asked whether this difference between L- and M-cones kinetics is an intrinsic or a stimulus-induced phenomenon. To address this issue, we calculated the impulse-response functions for M- and L-cones for 1-s periods at random time points throughout the NTSCI and used the time to peak (T2P) as an estimate of the cone response kinetics. T2P was constantly changing for both cone types throughout the NTSCI (Fig 1E). Most of the time, the L-cones were faster than the M-cones, but on some occasions, the M-cones were faster than the L-cones (e.g., arrowheads, Fig 1E). This suggests that the response kinetics of the cones were reacting to features of the NTSCI and did not represent an intrinsic difference between the L- and M-cones.

**Fig 2:**
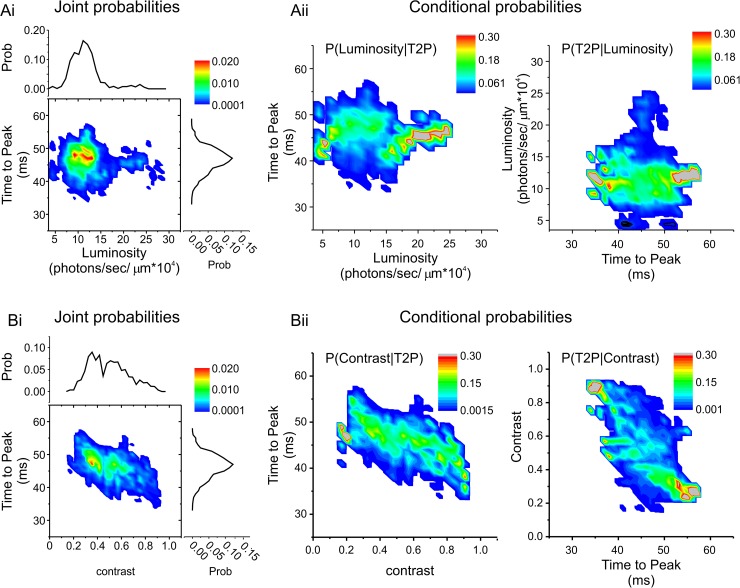
Joint and conditional probability maps. (Ai) The joint (heat map) and marginal (line graphs) probabilities for the “effective” local mean light intensity (luminosity) levels and the impulse-response time to peak (T2P) for the representative long- wavelength sensitive cone photoreceptor (L-cone) shown in Fig 1E. Also shown are the forward (Aii, left) and reverse (Aii, right) conditional probabilities for the same values shown in Ai. Overall, A indicates a statistical independence between the two variables. (Bi) The joint (heat map) and marginal probabilities (line graphs) for “effective” contrast levels and the impulse-response T2P for the representative L-cone shown in Fig 1E. Also shown are the forward (Bii, left) and reverse (Bii, right) conditional probabilities for the same values shown in Bi. Overall, B indicates a statistical dependency between the two variables. For both A and B, calculations were performed over 1-s periods starting at the locations shown in Fig 1E. The same analyses of the representative mid- wavelength sensitive cone photoreceptor (M-cone) shown in Fig 1E are given in S1B and S1C Fig. See Materials and Methods and S6 Fig for the definition of “effective” local mean light intensity and “effective” contrast. The data to generate this figure can be found in S1 Data.

Next, we assessed whether the change in kinetics of cones depended on the levels of luminance or contrast. To do this, we calculated the joint and conditional probabilities of the T2P with either the “effective” luminance or “effective” contrast level (see Materials and Methods and S6 Fig for “effective” level calculations). Fig 2A shows the relationship between the T2P and luminance for an L-cone, using 1-s periods at random time points. The random time points and T2P data are the same as shown in Fig 1E. T2P was largely statistically independent of luminance in both the joint (Fig 2Ai) and conditional probability (Fig 2Aii) distributions. On the other hand, T2P did covary with contrast as is demonstrated by the joint and conditional probabilities (Fig 2B). When contrast levels were higher, the T2P occurred earlier than when contrast was lower. This pattern was consistent for all four L- and all four M- cones we recorded from (see S1B and S1C Fig for the M-cone shown in Fig 1E). These results suggest that under naturalistic stimulus conditions M- and L-cone responses have faster kinetics during higher-contrast periods of the stimulus than when contrast levels are lower.

### Responses to artificial stimuli

The results shown in Fig 1 and Fig 2 demonstrate a novel and unexpected form of adaptation of cones: their response kinetics were faster during stimuli epochs with higher contrast levels than when contrast levels were lower. This result is unexpected, as previously, studies using white noise stimuli have indicated that cone response kinetics are unaffected by the level of contrast [12,13]. So far, we analyzed cone responses to the NTSCI. The advantages of such a stimulus are that it resembles the natural condition, where large fluctuations in light intensity can occur [3]. On the other hand, such a stimulus is rather erratic and not well suited for systems analysis. In the following sections, we study cone adaptation under more controlled conditions using artificial stimuli that are better suited for linear systems analysis.

Stimuli were generated by summing a range of sinusoids (sum of sinusoids, SoS) with different frequencies, equal amplitude, and randomized phase (see Materials and Methods), qualitatively similar to those previously used by Victor and colleagues [14]. Unlike classic stimuli such as white noise, these constructed stimuli retain a key feature typifying natural stimuli. By spacing the sine wave frequencies at approximately equal intervals on a log10 scale, much of the power of the stimuli came from the lower frequencies, similar to natural stimuli (S2 Fig).

**Fig 3:**
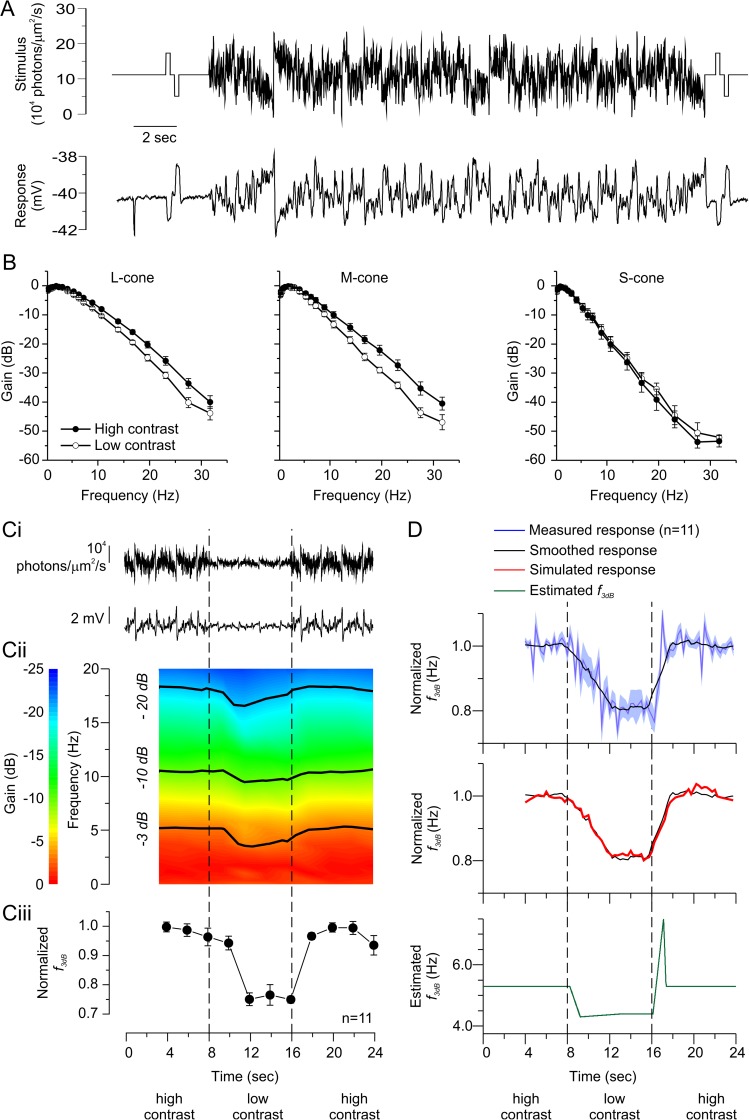
Contrast modulates the cone frequency response—sum of sinusoids (SoS) stimuli. (A) The light intensity pattern of the high-contrast SoS stimulus generated by summing 21 different frequency sinusoids with equal amplitude and randomized phase (upper trace, also see S2 Fig) and a long-wavelength sensitive cone photoreceptor’s (L-cone’s) response to this stimulus (lower trace). (B) Normalized frequency response amplitudes on a linear frequency scale for L- (left panel), mid- (middle panel), and short- (right panel) wavelength sensitive cone photoreceptors (M- and S- cones) in high- (filled symbols) and low- (open symbols) contrast conditions. Both L- and M-cones attenuated the higher frequency aspects of the stimuli less in high-contrast conditions than in low (see S2 Table for quantification). S-cones did not show this behavior. Instead, they heavily attenuated the higher frequencies of the stimulus in both contrast conditions. (Ci) a SoS stimulus generated by summing 18 sinusoids of different frequency sinusoids that switched from high to low temporal contrast at 8 s and then switched back to high contrast at 16 s (top trace) and a resulting L-cone response (bottom trace). Here and in all following experiments using switching stimuli, the results for L- and M-cones are pooled. (Cii) The mean spectrogram of 11 L- and M-cones shows that the cutoff frequencies at several gain levels (−3 dB (*f*_*3dB*_), −10 dB, and −20 dB, black lines) become lower when the stimulus switches from high to low contrast and become higher when the stimulus contrast switches back from low contrast to high. On average, switching contrast levels shifted *f*_*3dB*_ by 22% (Ciii) where each cone’s *f*_*3dB*_ during the first 0-to-4-s period was used to normalize each subsequent *f*_*3dB*_ (see text for statistics). (D) The time course of adaptation. The upper panel shows the measured cone response *f*_*3dB*_ every 250 ms (purple trace) and a smoothed version of the response (black trace). The middle panel compares the measured cone response *f*_*3dB*_ with the *f*_*3dB*_ of a stimulated cone response (red, see text and S4 Fig for details). In the lower panel, the *f*_*3dB*_ of the cone-derived filters used to generate the simulated cone response are given in 5-ms steps. Our simulation of the cone response suggests L- and M-cones start changing their frequency response characteristics within 100 ms of a contrast change. The adaptation process takes around 1 s to complete. Furthermore, it illustrates an asymmetry in the adaptation process. Data in B, Ciii, and D (upper) are shown as mean ± standard error of the mean (SEM). For B, frequency responses were generated using a 20-s window starting 2 s after the SoS stimulus onset. The stimulus was repeated multiple times (S1 Table) at one contrast level, and the average frequency response for each cone used. This procedure was then repeated at the other contrast level. The order of presentation was pseudorandom. See also S1 Table, S2 Table, S2 Fig, and S3 Fig. For C and D, the frequency responses were generated using 4-s windows every 2 s (C) or every 250 ms (D), and the data points shown correspond to the preceding 4-s interval. The stimulus was repeated 8 ± 1.1 times, and the average frequency response for each cone used. The data to generate this figure can be found in S1 Data.

Such stimuli are well suited for standard fast Fourier transform techniques, as the transfer functions derived from the cone responses to the SoS stimuli predicted 95 ± 0.7% (*n* = 36) of the cone’s light dependent structure (see Materials and Methods) (S3A and S3B Fig and S1 Table) [12]. Fig 3A shows a SoS stimulus (upper trace), and the resulting cone response is depicted in the lower trace. The frequency response curves for L- and M-cone voltage light responses to the high- and low-contrast versions of this stimulus are shown in Fig 3B (see S3C and S3D Fig for non-normalized impulse-response functions). Note that both stimuli had the same mean luminance. As for the NTSCI stimuli, *f*_*3dB*_ of L- and M-cones were the highest in the high-contrast condition and the lowest in the low-contrast condition (S2 Table). The slopes of the frequency response curves for L- and M-cones at higher frequencies were also shallower during high contrast than during low contrast (S2 Table). In addition, *f*_*3dB*_ and frequency response curve slopes for L-cones and M-cones did not differ from each other in either contrast condition (S2 Table). These results confirm our original observations with the NTSCI; L- and M-cone frequency response characteristics are not intrinsically different but are dependent on stimulus characteristics—in this case, the level of temporal contrast present. Interestingly, the frequency response of S-cones remained the same in the two contrast conditions (Fig 3B), and their *f*_*3dB*_ was lower and the slope of their frequency response curve steeper than for L- and M-cones in any contrast condition (S2 Table).

Next, we asked whether the change in frequency response characteristics of L- and M-cones was reversible within the same stimulus application. Cones were presented with an SoS stimulus that switched from high to low temporal contrast and back again (Fig 3Ci), and *f*_*3dB*_ of their frequency responses were determined (Fig 3Cii and 3Ciii). Since the behavior of L- and M-cones did not differ significantly, we pooled their data here and in all subsequent experiments using switching stimuli. On average, when going from high contrast to low contrast, *f*_*3dB*_ dropped by approximately 23% (Δ*f*_*3dB*_ = −1.2 ± 0.20 Hz, *n* = 11, *p* = 0.00012), and when the stimulus went from low to high contrast, it increased by approximately 22% (Δ*f*_*3dB*_ = 1.1 ± 0.19 Hz, *n* = 11, *p* = 0.00015) (Fig 3Ciii). These changes in *f*_*3dB*_ did not differ significantly from each other (*p* = 0.33), showing that adaptation in cones is reversible within the time course of this response.

### Time constant of adaptation

To study the time course of adaptation, cones were stimulated with the SoS contrast-switching stimulus, and *f*_*3dB*_ values were determined in 250-ms steps using frequency responses for 4-s periods with 93.75% overlap. The upper panel of Fig 3D shows the measured mean (±SEM) *f*_*3dB*_ of 11 L- or M-cones as a function of time during the switch from high contrast to low contrast and back again (blue trace). The curve was smoothed with an eight-point Savitzky-Golay filter (black trace). However, this result does not reflect the true time course of the change in *f*_*3dB*_. The 4-s time windows needed to accurately determine frequency responses when using this stimulus obscure the true time course. For instance, if *f*_*3dB*_ were to change at the same time as the contrast level switched, it would still appear as if the *f*_*3dB*_ change developed over the following 4 s as the analysis time window slides from one condition to the other. To estimate the true time course of adaptation, we simulated the adaptational process (S4 Fig). Cone filtering characteristics were systematically varied at different time points and convolved with the stimulus until we simulated a cone response that replicated the measured change in *f*_*3dB*_. Our best approximation of the cone mean *f*_*3dB*_ is shown in the middle panel of Fig 3D (red trace; smoothed with an eight-point Savitzky-Golay filter). The lower panel of Fig 3D shows *f*_*3dB*_ values used to produce this simulated response. When contrast levels switch from high to low, after a short delay, *f*_*3bB*_ steadily declines over about 1 s and then rebounds slightly over the next several seconds. When contrast switches back to high from low, after a short delay, the *f*_*3dB*_ rapidly increases over the next second, overshooting its final value, which it returns to over the next several hundred milliseconds. This analysis indicates that L- and M-cones start adapting relatively quickly after a change in contrast occurs and that the full adaptational process takes about a second to complete.

### Responses to SoS stimuli versus responses to white noise stimuli

**Fig 4:**
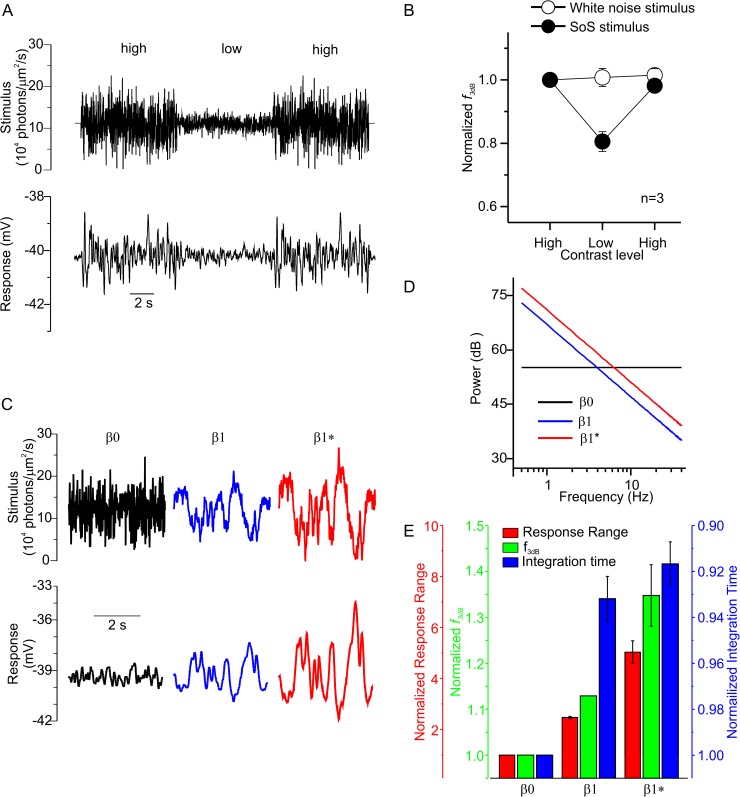
Adaptation depends on the frequency distribution of the stimulus. **(**A) A white noise (WN) stimulus that switches from high to low contrast and back again (upper) and a resulting long- wavelength sensitive cone photoreceptor (L-cone) response (lower). (B) The mean ± standard error of the mean (SEM) normalized *f*_*3dB*_ for three cones that experienced both the WN and the sum-of-sinusoids (SoS) (Fig 3Ci) contrast-switching stimuli. While *f*_*3dB*_ decreased when the SoS stimulus shifted from high contrast to low and increased when contrast switched back to high, *f*_*3dB*_ was unaffected when the WN stimulus switched between contrast levels. Panel C shows the β_0_ (black), β_1_ (blue), and β_1_* (red) stimuli in the upper panel, and an L-cone’s response to these stimuli is shown in the lower panel. The spectral power density of β_0_ was equal across all frequencies used, whereas it declined in a 1/f^1^ manner for β_1_ (D). β_1_* was a higher-contrast version of β_1_, generated by simply increasing the power of each frequency used by an equal amount. (E) The mean ± SEM response range (red), *f*_*3dB*_ (green), and integration time (blue) while under the β_0_, β_1_, or β_1_* stimuli. In each case, the data are normalized to the value obtained during the β_0_ condition. Seven mid- and long-wavelength sensitive cone photoreceptors (M- and L- cones) experienced both the β_0_ and the β_1_ stimuli, and five experienced both the β_0_ and the β_1_* stimuli. Note that the axes have been scaled so that the means for each response variable can be seen. The data to generate this figure can be found in S1 Data.

Previous studies using Gaussian white noise (WN) stimuli have not found adaptation in cones when contrast levels were varied [12,13]. We tested whether this was also true in our hands using WN stimuli that switched between high and low contrast (Fig 4A). To directly compare the cone responses, we presented three cones with both the SoS (Fig 3Ci) and WN (Fig 4A) switching contrast stimuli. Both stimuli were the same in terms of their maximum frequency (31.75 Hz), total stimulus power, and mean “stimulus” luminance and “stimulus” contrast level (see Materials and Methods and S6 Fig for “stimulus” contrast-level calculations). For these cones, under the WN condition, *f*_*3dB*_ remained unchanged when going from high to low (Δ*f*_*3dB*_ = 0.004 ± 0.0799 Hz, *p* = 0.97) contrast or from low to high (Δ*f*_*3dB*_ = 0.034 ± 0.029 Hz, *p* = 0.36) contrast (Fig 4B, open symbols). However, for the SoS stimulus, *f*_*3dB*_ decreased when contrast went from high to low (Δ*f*_*3dB*_ = 0.72 ± 0.149 Hz, *p* = 0.040) and increased when contrast went from low to high (Δ*f*_*3dB*_ = 0.65 ± 0.143 Hz, *p* = 0.046) (Fig 4B, closed symbols).

What stimulus difference might account for the absence of adaptation when WN is used? Since cones have a relatively long integration time, fast intensity variations will be averaged out. Hence, faster elements are increasingly “perceived” as a sustained light intensity with less variance until eventually, above the flicker fusion frequency, they simply appear as a sustained light stimulation. Consequently, stimuli like WN consisting largely of higher-frequency light intensity variations will have less “effective” contrast with which to drive cone responses than stimuli with a larger lower-frequency content (also see S2A–S2D Fig). To test this, we compared cone responses to stimuli in which we varied the power-frequency distribution (Fig 4C). Two stimuli had power-frequency distributions that declined in a 1/f^β^ fashion (β_1_ and β_1_*) and thus resembled natural scenes, and one was a WN version, as each frequency had equal power (β_0_) (Fig 4D). All stimuli were 4 s long, with a minimum and maximum frequency of 0.5 and 40 Hz, respectively. Both the β_0_ (Fig 4C, black trace) and β_1_ (Fig 4C, blue trace) stimuli had the same total power. For β_1_* (Fig 4C, red trace), we increased the power at each frequency of the β_1_ stimulus by 4 dB to maximize the light intensity variation delivered; hence, β_1_* is a higher-contrast version of β_1_.

The cone responses differed markedly under these different conditions. Fig 4C shows that the β_1_ stimulus induced a larger cone response than the β_0_ stimulus did. Across the seven cones tested under these conditions, the range of membrane potentials during β_1_ was more than twice that during β_0_ (2.5 ± 0.35 mV versus 6.1 ± 0.91 mV, *p* = 0.00035, Fig 4C and 4E), indicating that β_1_ was more effective at driving the cone response. The filtering characteristic and temporal response properties of cones also differed under these two stimulus components. Under the β_1_ condition, the cone *f*_*3dB*_ increased, and the temporal integration time decreased, compared to β_0_ (Fig 4E). These differences were even greater for five cones that received both the β_0_ and β_1_* stimuli. This suggests that the “effective” contrast “perceived” by the cones is lower in the β_0_ condition than in the β_1_ and β_1_* conditions. Indeed, when these stimuli are weighted by a function mimicking the temporal process of the phototransduction cascade (see Materials and Methods and S6 Fig), the resulting mean “effective” contrast levels for the β_1_ and β_1_* stimuli were approximately 2 and 3.5 times higher than for the β_0_ stimulus. These results indicate that (1) stimuli with a frequency distribution resembling natural stimuli drive cones more effectively and (2) adaptation was not found when using WN stimuli because the “effective” contrast in these stimuli was too low to modulate the cone membrane potential sufficiently to drive the adaptational process (also see S2E Fig).

### Underlying mechanism of adaptation in cones

**Fig 5:**
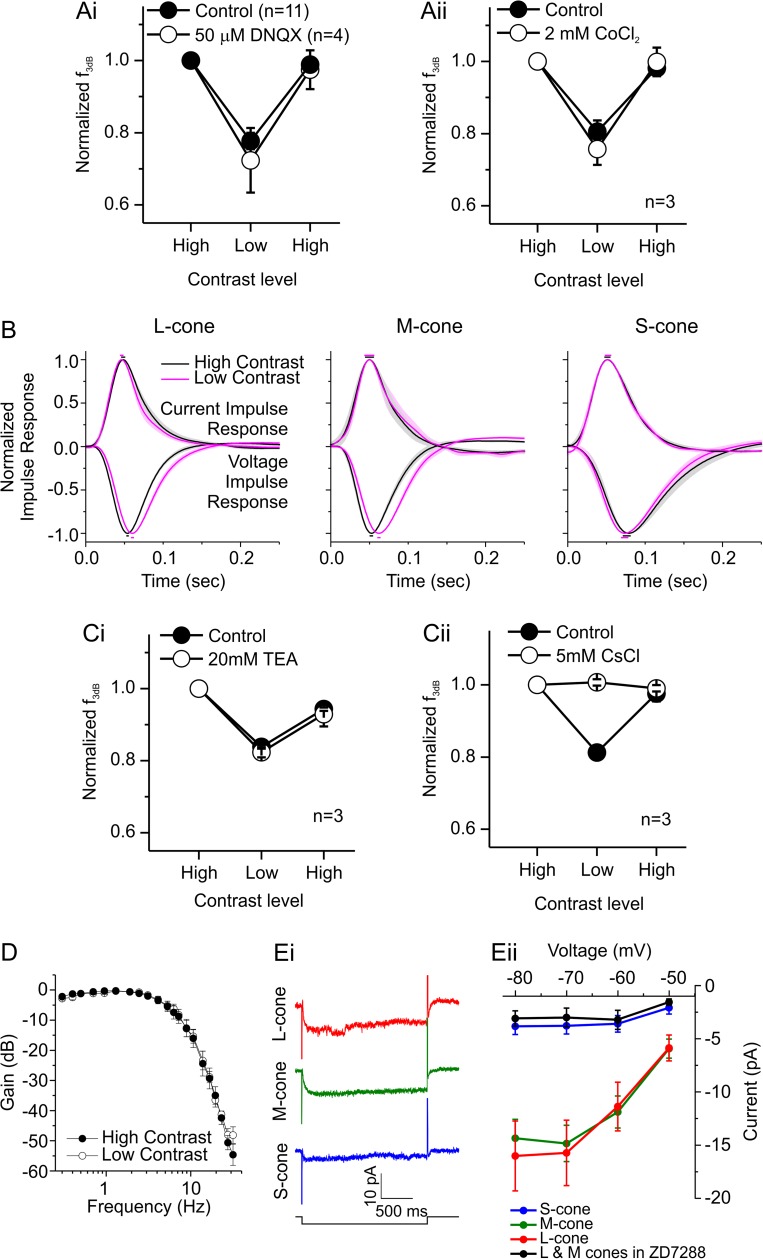
Localizing the adaptation mechanism. (A) The normalized *f*_*3dB*_ of long- and mid-wavelength sensitive cone photoreceptors (L- and M-cones) for contrast-level switches while blocking either cone input to horizontal cells with 6,7-dinitroquinoxaline-2,3-dione (DNQX, Ai) or cone synaptic transmission with CoCl_2_ (Aii). In both cases, switching contrast levels shifted *f*_*3dB*_ by the same proportion in both control and drug conditions, suggesting that adaptation is an intrinsic property of L- and M-cones. Note that the control data shown in Ai are for cells from Fig 3C, as stable light responses could not be maintained long enough for both control and DNQX conditions within the same cones. (B) Normalized current and voltage impulse-response functions of long-, mid-, and short-wavelength sensitive cone photoreceptors (L-, M-, and S-cones) when stimulated with two contrast levels (non-normalized results shown in S3C and S3D Fig). The current impulse responses are independent of contrast, while for the voltage impulse responses, high contrast speeds up L- and M-cone responses, but not S-cone responses (see S3 Table for quantification). These results suggest voltage-activated processes in the membrane of L- and M-cones allow them to adapt to contrast, and S-cones may lack these processes. (C) The normalized *f*_*3dB*_ of L- and M-cones when contrast levels switch and voltage-activated currents are blocked pharmacologically. Compared to control conditions, *f*_*3dB*_ shifted by the same percentage when in the presence of 20 mM of tetraethylammonium (TEA) (Ci), a blocker of the cone delayed rectifying potassium current (I_k_). However, *f*_*3dB*_ did not change with contrast when the hyperpolarization-activated inward rectifying current (I_h_) was blocked with 5 mM CsCl (Cii). These results indicate that I_h_ is an important component of the L- and M- cone adaptational mechanism. (D) The normalized frequency response amplitude of L- and M- cones when stimulated with two contrast levels while in the presence of 50 μM ZD7288, a specific I_h_ antagonist. These L- and M-cones did not adapt to contrast, confirming the important role of I_h_ in adaptation (see S2 Table for quantification). (E) To quantify I_h_ in L-, M-, and S-cones, the light response of the cones was suppressed by a 20-μm saturating spot of white light, and their membrane potential was clamped at −40 mV and then stepped for 2 s to potentials ranging from −80 mV to −50 mV in 10 mV increments. Individual cone responses to a potential step from −40 mV to −70 mV are shown in Ei. I_h_ was similar for the L- and M-cones (Eii, red and green symbols, *n* = 14 and 18, respectively) but was significantly smaller in S-cones (Eii, blue symbols, *n* = 9). In addition, the S-cone current approximately matched that found for L- and M-cones in the presence of 50 μM ZD7288 (Eii, black symbols, *n* = 5). Data are shown as mean ± standard error of the mean (SEM) except in Ei. In panel B, the color-coded bars above and below the impulse-response functions indicate the ±SEM in the time to peak. See text for statistics unless specified elsewhere. Panels A and C used the sum-of-sinusoids (SoS) contrast-switching stimulus shown in Fig 3Ci. Panels B and D used the SoS stimulus shown in Fig 3A and the same procedure described for Fig 3B. Experiments presented in panels A, C, and D were performed in current clamp. Number of stimulus repeats used (control, drug) in Ai: 8.0 ± 1.1, 8.5 ± 1.19; Aii: 7.7 ± 1.45, 7.7 ± 1.2; Ci: 5.7 ± 0.33, 6.3 ± 1.45; and Cii: 8.3 ± 0.88, 6.7 ± 0.88. For B, see S1 Table; for D: high contrast, 6.5 ± 0.67; low contrast, 6.8 ± 0.58. The data to generate this figure can be found in S1 Data.

What mechanism underlies the adaptation we find? First, we determined whether it is an intrinsic process of L- and M-cones by blocking either photoreceptor synaptic transmission with 2 mM CoCl_2_ or cone input to horizontal cells with 50 μM DNQX, a glutamate receptor antagonist. In both cases, adaptation was unaffected (Fig 5Ai and 5Aii) (for both DNQX and Co^2+^: 0.0001 < *p* < 0.045 for *f*_*3dB*_ changes going from high contrast to low or from low contrast to high in control and drug conditions). These results show that the adaptation process is cone intrinsic.

If changes in the phototransduction cascade were underlying this form of cone adaptation, then it should also be present in the photocurrent. To test this, we voltage clamped cones and determined their frequency response characteristics for two contrast conditions using the SoS stimuli shown in Fig 3A. Unlike Fig 3B, the frequency response curves of both the L- and M-cones fully overlapped in the two contrast conditions, showing that adaptation was absent in this condition (S5 Fig, S1 Table, S2 Table).

The difference between the voltage-clamp and current-clamp experiments is exemplified in Fig 5B, where impulse-response functions of cones for both high- and low-contrast conditions under both recording configurations are compared (see S3C and S3D Fig for un-normalized impulse-response functions). As expected, the voltage impulse-response function measured in current clamp of the L- and M-cones varied with contrast (Fig 5B, left and middle panel). Under low contrast, the T2Ps were longer, and the FWHMs broader (S3 Table). This did not happen in S-cones (Fig 5B, right panel, S3 Table). In comparison, the current impulse-response functions measured under voltage-clamp conditions for all cone types were the same under the different contrast conditions (Fig 5B, S3 Table). This result suggests that the adaptation depends on voltage-activated processes in the membrane of L- and M-cones and that such a component is largely missing in S-cones.

Which voltage-activated membrane process is critical for the L- and M-cone adaptation? The two most likely currents are (1) the delayed rectifying potassium current (I_K_) [15] and (2) the hyperpolarization-activated inward rectifying current (I_h_) [16–18]. Using the SoS contrast-switching stimuli (Fig 3Ci), *f*_*3bd*_ was determined for both high and low contrast in conditions when either I_h_ or I_K_ were pharmacologically blocked. In the following current-clamp experiments, current was injected in the cells to correct for drug-induced sustained changes to the light-adapted resting membrane potential. Twenty mM tetraethylammonium (TEA), a blocker for I_K_, reduced *f*_*3dB*_ in L- and M-cones by 54 ± 4.8% (*p* = 0.015, *n* = 3) but did not affect the adaptive changes (Fig 5Ci, *f*_*3dB*_ changes from high contrast to low or from low contrast to high; control, *p* < 0.007; TEA, *p* < 0.04). On the other hand, 5 mM CsCl, a blocker of both the potassium current and I_h_, reduced *f*_*3dB*_ in L- and M-cones by 28 ± 3.1% (*p* = 0.0010, *n* = 3) and prevented adaptation (Fig 5Cii, *f*_*3dB*_ changes from high contrast to low or from low contrast to high; control, *p* < 0.009; CsCl, *p* > 0.46). These results suggest that both I_K_ and I_h_ are speeding up the cone responses but only I_h_, and not I_k_, is involved in the cone adaptation we find. To confirm the contribution of I_h_ in the adaptation process, we tested whether adaptation occurred when I_h_ was blocked by 50 μM ZD7288, a specific I_h_ antagonist [18]. In this condition, neither *f*_*3dB*_ nor the slopes of the frequency response curves in the two contrast conditions were significantly different (Fig 5D, S2 Table). Combined, these experiments indicate that the mechanism underlying the M- and L-cone adaptation is strongly dependent on I_h_. This makes the adaptive process we identified a “hyperpolarization-activated adaptation,” which in turn emphasizes an essential property of the process: it is asymmetrical.

The absence of adaptation in S-cones (Fig 3B, S2 Table) suggests that they may have no or a smaller I_h_ compared to L- and M-cones. We tested this next. Whole cell current-voltage (I-V) relations were determined when the light responses of the cones were saturated with a small spot of light and cones were clamped at −40 mV and stepped to potentials ranging from −80 to −50 mV for 2 s. L-, M-, and S-cones developed a slow increase in an inward current (Fig 5Ei), a characteristic feature of I_h_ activation. The amplitude of I_h_ was determined by taking the difference between the peak of the initial current and the minimum current occurring within the next 1 s. This value was plotted as function of potential (Fig 5Eii). I_h_ did not differ significantly between L- and M-cones (0.66 < *p* < 0.97 for all potentials) but was significantly smaller in S-cones (for all potentials: versus L-cones, 0.0019 < *p* < 0.012; versus M-cones, 0.00005 < *p* < 0.0014). Inhibiting I_h_ with ZD7288 reduced I_h_ in L- and M-cones to the level found in S-cones (Fig 5Eii, 0.43 < *p* < 0.76 for all potentials). These results indicate that S-cones do not adapt like L- and M-cones because they lack I_h_, corroborating its importance as a critical membrane component required for the form of adaptation we find.

### Kinetic properties of cones

**Fig 6:**
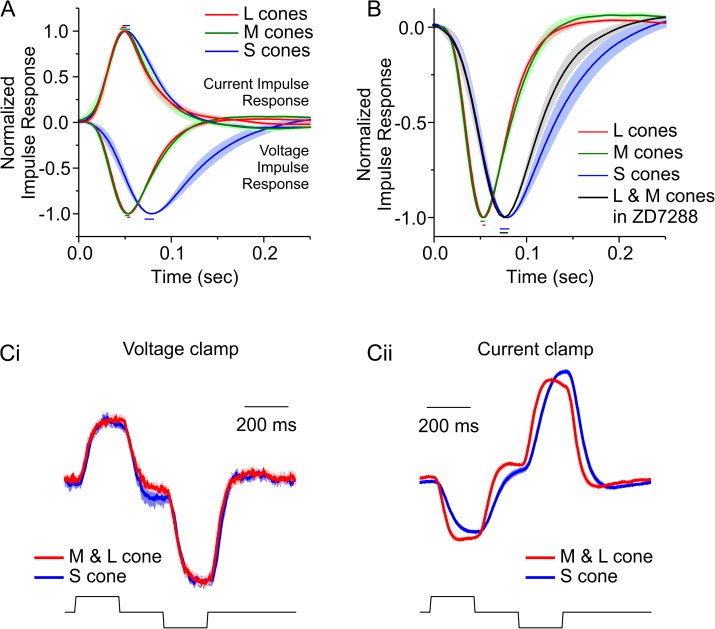
Comparison of light responses under the voltage- and current-clamp conditions of long-, mid-, and short- wavelength sensitive cone photoreceptors (L-, M-, and S-cones). (A) L-, M-, and S-cone current and voltage impulse-response functions during high contrast. Current impulse responses were equal for all cone types (see S3 Table for quantification). (B) Voltage impulse responses during high contrast for L-, M-, and S-cones and for the pooled result of L- and M-cones in the presence of 50 μM ZD7288. When I_h_ is antagonized with ZD7288, the voltage light responses of L- and M-cones slows and closely resembles that of S-cones (see S3 Table for quantification). Panels A and B suggest that cone membrane properties (membrane capacitance) slow the light response and that voltage-gated currents such as I_h_ speed up the light response of L- and M-cones. Since S-cones lack a prominent I_h_ (Fig 5Eii), their voltage light responses remain slow. (C) When stimulated with a simple brief-light step stimulus, while the current responses of all three cone types had similar kinetics (Ci), the voltage response of S-cones was considerably slower (Cii). Panels A and B used the sum-of-sinusoids (SoS) stimulus and procedure described in Fig 3A and 3B. The L-, M-, and S-cone data are the same as shown in Fig 5B; for the ten L- and M-cones in ZD7288, the stimulus repeated 5.7 ± 0.56 times. Data for panel C were 0 to 1 normalized before averaging. All data are shown as mean ± standard error of the mean (SEM); the color-coded bars above and below the impulse-response functions indicate the ±SEM in the time to peak. The data to generate this figure can be found in S1 Data.

The comparison of voltage-clamp and current-clamp data also demonstrates another important role of I_h_. The current impulse-response functions of all cone types in low-contrast conditions were faster and narrower compared to the voltage impulse-response functions, and the current impulse-response functions of the different cone-types did not differ from each other (Fig 6A, S3 Table). This indicates two points: (1) The phototransduction cascade of all cone types have the same kinetic properties, and (2) membrane properties of the cones, most likely the membrane capacitance, slow down the responses under current clamp. Fig 6B shows that I_h_ speeds up the kinetics of the cone responses. When I_h_ is blocked by ZD7822, the voltage impulse-response functions of L- and M-cones became as slow as those of the S-cones (S3 Table). This slowing down of the response is prominent in L- and M-cones and absent in S-cones since the latter have no I_h_ (Fig 6A, S3 Table).

This difference between the S-cone kinetics and the L- and M-cone kinetics was also evident when cones were stimulated with light flashes of 200 ms, which were 50% brighter or 50% dimmer than the mean luminance of the SoS stimuli. The responses under voltage clamp overlapped for the L-, M-, and S-cones (Fig 6Ci), but under current clamp, S-cone responses were substantially slower (Fig 6Cii). Combined, these results indicate that (1) the kinetics of the phototransduction cascade in L-, M-, and S-cones are equal; (2) the membrane properties of cones, most likely the membrane capacitance, slow down the kinetics of their voltage light responses considerably; and (3) in L- and M-cones, but not in S-cones, I_h_ speeds up the cone voltage light responses such that they approach the cone photocurrent responses. Furthermore, the presence of I_h_ allows the cone kinetics to be adaptive.

## Discussion

In this paper, we show that L- and M-cones can adaptively alter their frequency response characteristics via a phototransduction-independent mechanism that depends instead on membrane hyperpolarization: “hyperpolarization-activated adaptation.” The hyperpolarization-activated current, I_h_, is a critical component of this mechanism, as blocking it pharmacologically prevented the adaptive response. Corroborating the important role of I_h_ in this adaptive process, S-cones did not demonstrate hyperpolarization-activated adaptation, and they also lacked a prominent I_h._ As this form of cone adaptation is dependent on voltage-activated processes, it is only apparent when the cone membrane potential is sufficiently modulated—for example, under naturalistic stimulus conditions when temporal contrast is high. Thus, these results suggest that under naturalistic stimulus conditions, the relative activation of I_h_ and the resulting changes in the kinetics of the cones follow temporal contrast.

### The action of I_h_

How might I_h_ change the frequency characteristics of cones in different temporal contrast conditions? I_h_ is known to affect the kinetic properties of neurons [19,20]. Experiments in which I_h_ was blocked, either pharmacologically or by knocking out the hyperpolarization-activated cyclic nucleotide-gated (HCN) channels mediating I_h_, showed that I_h_ makes rod light responses more transient, changes the filter characteristics of rods from low pass to band pass, and increases the cutoff frequency [18,21,22]. Similarly, increased activation of I_h_ moves the peak of the cones' transfer function to higher frequencies, in this way extending the operational frequency range of cones [18].

I_h_ can affect a neuron in various ways. Activation of I_h_ by hyperpolarization decreases the input resistance, depolarizes the resting membrane potential, and speeds up the kinetics of neurons [19,20,23,24]. Activation of I_h_ also leads to a reduction of low frequencies because of the channel’s slow kinetics. On the other hand, the increase in membrane conductance due to the activation of I_h_ leads to a decrease of the membrane time constant because the membrane capacitance can be discharged faster and thus speeds up responses [18]. These effects would lead to a reduction in gain mostly at low frequencies.

However, we also find that a moderate increase in gain at high frequencies can occur (S3C Fig), indicating that I_h_ may also modulate a mechanism for an overall gain increase. Although we have shown that the activation of I_h_ is essential for the form of adaptation we find for L- and M-cones, we cannot discount the possibility that other factors contribute to this overall gain increase. These additional factors might include second messenger modulation of the I_h_ activation potential, local changes in ion concentration near the cone membrane [25], and indirectly activated ion channels [26]. In addition, I_h_ becomes faster when the membrane potential is hyperpolarized [27].

I_h_ seems to be essential for the fast response kinetics of cones. When I_h_ is not active, the impulse-response functions of cones under voltage clamp are faster than under current clamp (S3 Table), indicating that the membrane capacitance of the cones slows down the voltage light responses considerably. I_h_ counteracts this such that the L- and M-cone responses under current clamp in high contrast are about equally fast as under voltage clamp (Fig 6A, S3 Table). This does not happen in S-cones since they lack a prominent I_h_ (Fig 5E). In addition, I_K_ also helps to speed up the cone light response.

The adaptation we have found in L- and M-cones may therefore reflect the activation of I_h_. This may be a consequence of larger cone membrane potential fluctuations around the resting membrane potential in high-contrast conditions. As I_h_ becomes larger and activates faster at more hyperpolarizing potentials [19,27], it will be larger in the high-contrast condition and smaller in the low-contrast condition. When I_h_ is more activated, it will speed up the light response. In low-contrast conditions, I_h_ will be smaller, so the voltage response will remain slow, dominated by the passive low-pass filter properties of the membrane.

Once activated by hyperpolarization, I_h_ can remain active for hundreds of milliseconds, even if the membrane potential has since depolarized [28]. During high-contrast conditions, I_h_ remains activated since sufficiently hyperpolarizing events occur while I_h_ is still active from previous events and cone response kinetics therefore remain faster. However, the situation is different for a luminance step in which the cone experiences a sustained increase in light intensity. Here, I_h_ activation is transient as the additional adaptation processes of the phototransduction cascade come into play. Over time courses of hundreds of milliseconds to seconds, the phototransduction cascade becomes increasingly adapted. This depolarizes the cone membrane potential back towards its preluminance step potential [29] to a far greater extent than any depolarization resulting from I_h_ activation. Consequently, I_h_ activation will reduce, and any increase in cone kinetics that occurred at the beginning of the luminance step will disappear. Hence, the adaptive responses to dynamic and static changes in light intensity are different: high-contrast-like conditions will keep I_h_ activated, whereas luminance-step-like conditions will not.

Is hyperpolarization-activated adaptation similar to the dynamic light adaptation proposed by Clark et al. [30]? Their phenomenological model consists of two kernels that extend over similar time scales, but one is broader and delayed relative to the other. The two kernels combine to produce an “effective” kernel in which the time scale and dynamics are dependent on the recent stimulus history. Using this model, they were able to reproduce many features of cone responses, including previously unreported gain changes in cone responses to a WN stimulus. Although we were able to fit their model to our WN data, despite our best efforts, we were unable to adequately fit their model to our SoS data. The dynamic light adaptation model overestimated the cone response to decrements in light intensity and underestimated the time course for these changes under the SoS conditions.

One potential reason that the model of Clark et al. [30] may not adequately simulate our results is that it uses one nonlinearity to describe both the asymmetry between cone responses to light increments and decrements (Fig 6C) and a change in response kinetics under differing light intensity. However, for our results we find that this asymmetry and the changes to the kinetics of cone response have different origins. For example, under voltage clamp while we find that the asymmetry between cone responses to light increments and decrements is present (Fig 6C), the response kinetics are unaffected by stimulus contrast (Fig 5B, S3 Fig, S5 Fig). Hence, these two processes have a different origin and therefore cannot be adequately described by one nonlinearity. This illustrates the limits of simplistic linear/nonlinear modelling.

### The time constant of hyperpolarization-activated adaptation

How does the time constant of hyperpolarization-activated adaptation compare with other known adaptational processes in the retina? Light adaptation occurs throughout the animal kingdom and is active at many levels in the visual system, occurring over multiple scales in time and space. For example, the retinal cone pathway adapts to small changes in luminance at the bipolar to ganglion cell synapse, whereas larger changes induce adaptation within the cones themselves that occur over time scales ranging from tens of milliseconds to minutes [2,30–32]. Similarly, some mechanisms for temporal contrast adaptation are relatively fast, in the hundreds-of-milliseconds range or less [14,33], whereas others are relatively slow, on the order of several seconds [13,34]. The spatial extent ranges from the size of ganglion cell receptive field subunits to the whole retina [33]. The hyperpolarization-activated adaptation we find for L- and M-cones appears to be one of the retina’s faster mechanisms. It seems to begin within 100 ms of an abrupt change in the modulation depth of the membrane potential, as happened when our SoS stimuli switched from one contrast condition to another, and once started, the adaptational process continues over about a second. Hyperpolarization-activated adaptation does not seem to be symmetrical; the time course is different when the membrane modulation depth decreases (i.e., going from high to low contrast) versus when it increases (i.e., going from low to high contrast). Interestingly, optimal adaptation to nonstationary variance has been suggested to have asymmetric dynamics, as an abrupt increase is more readily detectable than an abrupt decrease [35,36].

### What is hyperpolarization-activated adaptation?

So far, we have shown that L- and M-cones possess a novel form of adaptation: hyperpolarization-activated adaptation. However, is this novel form of adaptation a type of luminance or contrast adaptation?

In Fig 2 (and S2 Fig), we show that cones change their kinetics in response to variations in contrast and not to luminance. Therefore, one might be tempted to call hyperpolarization-activated adaptation a form of contrast adaptation. However, we will not do so for the following reason. Contrast can be positive or negative. Positive contrast corresponds with cone hyperpolarization, while negative contrast corresponds to depolarization. A true contrast adaptation mechanism should be activated by both positive and negative contrast. Since the mechanism we have identified is hyperpolarization-activated adaptation, it can only be activated by positive contrast and is therefore not a true contrast adaptation mechanism. However, it should be noted that previous investigations into mechanisms of contrast adaptation at various retinal locations have not always used such a strict definition. Often the mechanism under study is only invoked by changes in the “preferred” contrast, such as positive contrast for ON-bipolar cells and negative contrast for OFF-bipolar cells [37–40].

Does that mean that the mechanism we have identified is a form of luminance adaptation? Light stimulation will hyperpolarize cones, which activates I_h_ and induces adaptation. Thus, one might be tempted to call hyperpolarization-activated adaptation a form of luminance adaptation. However, as we describe above, I_h_ is transiently activated by sustained changes in light intensity. Hence, the hyperpolarization-activated mechanism we describe cannot remain adapted to a luminance level over longer time periods. It is a transient luminance adaptation mechanism. Indeed, this situation is reflected in the results found for the NTSCI. Hyperpolarization-activated adaptation did not correlate with the mean luminance over 1 s periods, whereas it did correlate with contrast. Therefore, we cannot call this adaptive mechanism a form of true luminance adaptation.

Fast luminance adaptation has been described and can occur in cones on time scales from tens to hundreds of milliseconds [30–32]. However, extensive modelling [41] and direct cone measurements [32,42] indicate that fast luminance adaptation arises from the phototransduction cascade, and hence, they are distinct from the form of adaptation we find.

Is the hyperpolarization-activated adaptation we describe even a form of light adaption? Light adaptation as the name implies depends on light, but hyperpolarization-activated adaptation does not. It is fully determined by the modulation of the cone membrane potential. Therefore, in the most exacting sense of the name, the form of cone adaptation we find is not truly light adaptation, but it can be induced by light stimuli. In essence, the mechanism we have identified is rather difficult to classify within the boundaries of the existing and well-known terminology. It is neither luminance, nor contrast, nor even light adaptation within their strictest definitions. This raises the question of how best to name these adaptational phenomena. Do we name them according to the stimulus feature driving them best or by stricter criteria? Here, we have chosen the latter and name the form of adaptation we find “hyperpolarization-activated adaptation.”

Here, we have shown that the main aspect of the stimulus that activates hyperpolarization-activated adaptation in a natural condition is the ability of a stimulus to engage and modulate the cone membrane potential. In other words, the more one stimulus condition can hyperpolarize the cone membrane potential away from the mean membrane potential and modulate I_h_ compared to another stimulus condition, the bigger the relative changes in kinetic properties of the cone will be. Hence, under natural conditions, cone responses will be faster during prolonged periods when the dispersal of light intensities are broader, especially if skewed towards higher values, than when the dispersal is narrower. This dispersal could be estimated by the variation of intensities occurring around the prolonged period’s mean value (contrast) as we have done. However, it could just as easily be described by the distributions of mean intensities calculated over shorter time windows (luminance) within each period. As low-frequency-dominated stimuli like natural scenes have long autocorrelation times, distinguishing between these two measures of dispersal becomes increasingly difficult as the time window used to perform the calculations becomes shorter. Consequently, while we cannot say that the adaptive response we find is exclusively a form of contrast or fast and transient luminance adaptation, we can say that when measured as we have done, cone responses speed up in high-contrast conditions and slow down in low-contrast conditions and thus behave like a contrast adaptation mechanism.

### Why did we find an adaptive response with contrast, whereas others did not?

How does hyperpolarization-activated adaptation relate to known contrast adaptation mechanisms? Adaptation to temporal contrast is thought to only occur in higher-order neurons such as bipolar cells, amacrine cells, and ganglion cells [13,40,43,44]. However, as we show here, this is not entirely the case, as contrast changes can also induce an adaptive response in L- and M-cones. What could be the reason for this different result? Previous studies based their conclusions on experiments using WN stimuli, which were confirmed in the present paper (Fig 4). However, such stimuli differ substantially from the naturalistic and artificial stimuli that induced hyperpolarization-activated adaptation here.

Natural stimuli typically have long-term serial correlations, and as their power spectra decreases with 1/f^β^ (0.7 < β < 3), they predominately contain lower frequencies [3,4], whereas WN stimuli contain no serial correlations, and as the power of each frequency is equal, WN signals mostly consist of higher frequencies. As cones have relatively long integration times, the high-frequency component of the WN stimulus will be perceived by the cone as a sustained light stimulus, thereby reducing the “effective” contrast (S2 Fig, S6 Fig). Consequently, stimuli like WN that consist primarily of higher frequencies will not modulate the membrane potential of cones as effectively as stimuli like natural stimuli that largely consist of lower temporal frequencies, even though they may be equal in terms of both the total photon number and variance (Fig 4, S2E Fig). If the membrane potential of the cone is not sufficiently modulated, then I_h_ will not activate enough to cause measurable hyperpolarization-activated adaptation. In the studies of Rieke [12] and Baccus and Meister [13], the high-contrast noise stimuli generated membrane potential changes in cones that were similar to or smaller than those found by us in SoS low-contrast conditions (S2E Fig). Hence, one should determine how effective a stimulus is at modulating the membrane potential of cones, as this will determine their adaptational state. Here, we estimated this using the “effective” contrast metric. Previous studies have also noted that “effective” contrast is a better measure of a stimulus’s ability to engage other neurons in the visual system [14,45–47] and lateral geniculate nucleus (LGN) neurons [48] than “stimulus” contrast is.

That WN stimuli cannot induce hyperpolarization-activated adaptation in cones whereas it can induce adaptive changes in later visual system neurons indicates that independent contrast adaptation mechanisms exist at different stages of the visual system. These contrast adaptation processes have been studied mostly with WN stimuli. Since cones respond better to naturalistic and “natural-like” stimuli, as we have shown in this paper, it is likely that these higher-contrast adaptation mechanisms might become more pronounced if stimuli that at least preserve features of the natural world are used. Presumably, the higher effective contrasts delivered by WN stimuli, when band limited to lower frequencies (e.g., 10 Hz) so that the waveforms of light intensities match the operational range of photoreceptors, would also engage these adaptation mechanisms more fully.

Thus, in the broader perspective, it would be very interesting to re-examine temporal contrast adaptation in neurons throughout the visual pathway using stimuli that can deliver high levels of effective contrast, like natural stimuli. As sensory systems have evolved to process natural stimuli, it is highly likely that the whole visual pathway is optimized to process stimuli with spectral power distributions and serial correlations similar to those occurring in natural scenes. Indeed, sensory neurons display different response kinetics and filter characteristics as well as increased encoding efficiency when natural stimuli are used. Responses to classic stimuli are often poorly predictive for responses to natural stimuli, and as we show here, some response properties are only apparent when naturalistic stimuli are used [49–55].

Finally, with regards to the gain changes that are typically associated with contrast adaptation, what we find for cones is unique. Later visual neurons typically adapt to an increase in contrast by reducing their gain and integration time, processes that can occur independent of each other [13,40,43,44,56]. However, the gain at higher frequencies of the cone response increased, not decreased, with increased contrast levels. This increased higher-frequency gain is entirely consistent with a reduced integration time.

### Functional consequences

How might hyperpolarization-activated adaptation improve the performance of cones? Van Hateren [57] proposed that sensory neurons will act as adaptive filters that maximize the level of information in different SNR conditions. In high-SNR conditions, they will integrate the incoming signal over a shorter period of time and thereby transmit more information. However, in low-SNR conditions, they will increase their integration time, effectively sacrificing the higher-frequency components of the stimulus to reduce the higher-frequency noise contained in their responses. In this way, the sensory neurons restrict and adjust their response range to frequencies at which the signal can be reliably transmitted.

In this paper, we have shown that in a natural scene L- and M-cones adapt independently to contrast via a process called hyperpolarization-activated adaptation. The consequence is that within the same scene, cones of different types and of the same type at different retinal locations experience distinct visual environments and adjust their output accordingly. For instance, an M-cone looking at foliage experiences a low-contrast environment, while an L-cone looking at a red flower in the foliage experiences a high-contrast environment. In such a scene, L- and M-cones adapt their filtering properties such that they may sample their “cone-specific” scene more optimally.

The finding that S-cones are slower than L- and M-cones and do not adapt to temporal contrast might reflect the ecological properties of the short-wavelength environment. In natural scenes, the short-wavelength environment has a narrower distribution of contrast levels than the mid-wavelength environment [58], making it less important for S-cones to adapt to the temporal contrast than for L- and M-cones. The results presented in this paper show that L- and M-cones use hyperpolarization-activated adaptation to extract the most reliable information from their “cone-specific” scene.

## Materials and methods

### Isolated retina preparation and electrophysiology

All animal experiments were carried out under the protocol NIN10.31 issued by the ethical committee of the Royal Netherlands Academy of Arts and Sciences acting in accordance with the European Communities Council Directive of 24 November 1986 (86/609/EEC). Goldfish, *Carassius auratus*, were killed, and the eyes were enucleated, with the retina isolated under infrared illumination with the aid of IR viewers, placed photoreceptor side up in a recording chamber (volume: ~300 μl, model RC-26G, Warner Instruments) mounted on a Nikon Eclipse 600FN microscope, secured under a tissue harp, and continuously superfused (1.5 ml.min^−1^) with oxygenated Ringer’s solution at room temperature (20°C). The preparation was viewed on an LCD monitor by means of a 60× water-immersion objective (N.A. 1.0), a CCD camera, and infrared (λ > 800 nm; Kodak wratten filter 87c, United States) differential interference contrast optics. Cone photoreceptor outer segments were visually inspected for damage, and whole cell recordings from undamaged cones with resting membrane potentials between −35 and −45 mV (mean: −39.6 ± 0.73 mV) were made under voltage or current clamp (series resistance 25–40 MΩ). The current light response was measured by voltage clamping cones at their resting membrane potential, whereas the voltage light response was measured by current clamping cones. When current clamped, holding currents were only applied when CoCl_2_, CsCl, or TEA-Cl were in the perfusate in order to restore the light-adapted membrane potential back to its original value. The data were not corrected for junction potentials.

### Solutions

Ringer solution consisted of the following (in mM): 102.0 NaCl, 2.6 KCl, 1.0 MgCl_2_, 1.0 CaCl_2_, 28.0 NaHCO_3_, 5.0 glucose continuously gassed with 2.5% CO_2_ and 97.5% O_2_ to yield a pH of 7.8 (osmolarity 245–255 mOsm). When using TEA, the NaCl concentration was equimolar reduced to maintain the chloride equilibrium potential. Pipette solution was made fresh every 2–3 days and contained (in mM) 96 K-gluconate, 10 KCl, 1 MgCl_2_, 0.1 CaCl_2_, 5 EGTA, 5 HEPES, 5 ATP-K_2_, 1 GTP-Na_3_, 0.1 cGMP-Na, 20 phosphocreatine-Na_2_, and 50 units ml^−1^ creatine phosphokinase, adjusted with NaOH to pH 7.27–7.3 (osmolarity 265–275 mOsm). All chemicals were supplied by Sigma-Aldrich (Zwijndrecht, the Netherlands), except for ZD7288 (Tocris Biosciences, Bristol, United Kingdom).

### Electrodes and recording setup

Patch pipettes (resistance 8–12 MΩ, PG-150T-10; Harvard Apparatus, Holliston, Massachusetts) were pulled with a Brown Flaming Puller (Model P-87; Sutter Instruments Company). Pipettes were placed in a PCS-5000 micromanipulator (Burleigh Instruments, Union City, California), connected to an Axopatch 200A patch clamp amplifier (Molecular Devices, Sunnyvale, California, four-pole low-pass Bessel filter setting: 1 kHz). Data were digitized and stored with a PC using a CED 1401plus AD/DA converter at 2 kHz sampling frequency using Signal software (v. 3.07; Cambridge Electronic Design [CED], Cambridge, UK) to acquire data, generate voltage command outputs, and drive light stimuli.

### Light stimuli

The light stimulator consisted of a homemade LED stimulator based on a three-wavelength high-intensity LED (Atlas, Lamina Ceramics, Westhampton, New Jersey, US). The peak wavelengths of the LEDs were 624, 525, and 465 nm, respectively, with bandwidths smaller than 25 nm. An optical feedback loop ensured linearity. The output of the LEDs was coupled to the microscope via fiber optic light guides. Stimuli were projected onto the retina via a 20-μm light spot focused on the cone outer segment though a 60× water-immersion objective at a presentation rate of 166.67 Hz for the NTSCI and 200 Hz for all other stimuli. White light consisted of equal quantal output of the three LEDs.

#### Light intensities

The mean light intensity for all stimuli was at photopic light levels for goldfish [59,60]. The mean light intensities of the NTSCI for L- and M-cones were 1.13 * 10^4^ and 1.24 * 10^4^ photons/μm^2^/s, respectively. All other stimuli used had a mean light intensity of 1.20 * 10^4^ photons/μm^2^/s.

#### Luminosity and temporal contrast

Cones have relatively long temporal integrations times, so faster variations in light intensity become increasingly less available to cones. Hence, we first calculated the “effective stimuli” presented to cones by weighting stimuli with an estimate of the temporal integration time of the phototransduction process (S6 Fig). Local mean light intensity values and temporal contrast were then calculated. These values are termed the “effective” luminance and the “effective” contrast. For any given stimulus segment, its local mean intensity was simply the average value, and its temporal contrast was its standard deviation divided by its average. We also performed the same local mean light intensity values and temporal contrast calculations prior to generating the “effective stimuli.” These values are termed “stimulus” luminosity and “stimulus” contrast.

When using such a contrast measure, the level and range of contrast values of a stimulus are contingent on the time interval over which they are calculated [61]. As such, we have refrained from giving an actual value to the contrast levels delivered by our various artificial stimuli. Rather, we talk in terms of high and low. This is because while we cannot say for certain exactly what contrast levels cones “perceived,” we can say that when calculated over a wide range of time windows, a broader range of contrasts, with higher median values and a greater portion of higher values, was delivered by our high-contrast stimuli (S6 Fig). For the NTSCI data shown in Fig 2 and S1B and S1C Fig, where actual values are necessary, “effective” contrast and “effective” luminance values were calculated for 1-s periods. The means of these values were used when describing the difference mean contrast and luminosity conditions for the two cone-specific stimuli. Using 1-s periods to generate the contrast and luminosity values was an arbitrary choice and largely based on the shortest time period that gave a reasonable frequency resolution in the Fourier domain.

#### NTSCI

The NTSCI was a 40-s sequence of the natural stimulus “the flower show” from van Hateren's natural-stimulus collection [9]. It was preceded and followed by a 4.5-s period of stimulus mean-intensity white light.

#### SoS stimuli

The SoS stimuli used are shown in Fig 3A and 3C. Respectively, they were generated by adding a mean intensity to the sum of the following 21 or 18 sinusoidal wave frequencies (in Hz)—Fig 3A: 0.3, 0.4, 0.5, 0.7, 0.95, 1.3, 1.9, 2.45, 3.1, 4.1, 5.3, 6.3, 7.2, 8.9, 10.7, 13.9, 16.7, 19.6, 23.1, 27.5, 31.7; and Fig 3C: 0.5, 0.75, 1.25, 1.75, 2.75, 3.25, 4.25, 4.75, 5.75, 7.25, 9.25, 10.75, 14.75, 17.75, 20.75, 24.25, 27.25, and 31.75. In addition to the reasons given in the results and S2 Fig, the frequencies used were also chosen to minimize higher-frequency harmonics of lower frequencies, spectral leakage between measured frequencies, and contamination by equipment noise (typically whole number frequencies).

#### WN stimulus

Like the SoS stimulus shown in Fig 3Ci, the WN stimulus switched from an 8-s period of high contrast to an 8-s period of low contrast and then back to another 8 s of high contrast (Fig 4A). The same 8-s series of Gaussian fluctuations of light intensities was used for both high-contrast components, and a scaled version was used for the low-contrast component. The stimulus was sharp low-pass filtered at 31.75 Hz, and the resulting high- and low-contrast components had the same total stimuli power, “stimulus” luminance, and “stimulus” contrast levels (4-s time windows) as the high- and low-contrast SoS stimuli, respectively.

#### Beta stimuli

Beta stimuli were 4 s long. β_0_ and β_1_ were constructed by first adding a random phase value to the frequency response magnitude data shown in Fig 4D. For β_1_*, the magnitude component of the β_1_ frequency response was increased by 4 dB. The frequencies used ranged from 0.5 to 40 Hz in 0.25 Hz increments. Each sequence was zero padded out to 200 Hz and converted to a time series by inverse Fourier transformation of a two-sided version of this padded sequence. When summed across the frequencies used, the total stimulus power was equal for the β_0_ and β_1_ stimuli, as were the “stimulus” luminance and “stimulus” contrast levels when calculated for the 4-s periods.

### Frequency response

Using Welch's averaged periodogram method [62], frequency responses *F*_*sr*_ *(f)* (Eq 1) were calculated as the quotient of the cross power spectral density of *S (f)* and *R (f)* and the power spectral density of *S (f)*:

$$F_{sr}(f)=\left\langle \frac{S^{*}(f)R(f)}{S(f)S(f)}\right\rangle ,$$ (1)

where * is the complex conjugate and < > denotes averaging over multiple stimulus presentation repeats. Where frequency response curves are used, only the magnitude data are shown. Frequency responses were calculated using the following parameters:

1. *NTSCI*: The cone-specific stimuli were up-sampled to 1 kHz, and the subsequent analysis was performed at this sample rate. For Fig 1C and 1D (and S1A Fig), frequency responses were calculated for 5-s nonoverlapping periods of the stimulus and corresponding cone responses, which were then averaged. Each frequency response used a 5-s discrete Fourier transform length, windowed with eight equal-length periodic Hamming windows that overlapped by 50%. For Fig 1E, Fig 2, and S1B and S1C Fig, the same frequency response method was used, but the length of the stimulus, corresponding cone responses, and discrete Fourier transform periods were 1-s periods. These 1-s stimulus and corresponding cone response periods were pseudorandomly resampled 10,000 times. In approximate 35% of cases, convolving the impulse-response function with the stimulus was poorly predictive of the cone response, typically during periods in which the stimuli were largely quiescent. We therefore restricted our analysis to periods in which the correlation coefficient between the cone response and the predicted response of the impulse-response function exceeded 0.75.
2. *SoS stimuli*: Discrete Fourier transform lengths were set such that each sine wave used to generate the SoS stimuli had completed a whole number of cycles and their frequencies were resolved. No windowing method was needed. The stimulus generated with 21 sinusoids needed a 20-s Fourier transform length (or multiples of). The stimulus was up-sampled to 1 kHz, and the analysis performed on 20-s lengths of the stimulus and cone response at a 1 kHz sample rate. The stimulus generated with 18 sinusoids required Fourier transform lengths of 4 s (or multiples of). The analysis was performed on either 4-s or 8-s lengths of the stimulus and cone response at a 200 Hz sample rate.
3. *WN stimuli*: 8-s lengths of the stimulus and cone response were used. The Fourier transform length was 8 s using 97.5% overlapping 1-s rectangle windows, performed at a 200 Hz sample rate.
4. *Beta*: 4-s lengths of the stimulus and cone response were used. The Fourier transform length was 2 s using 75% overlapping 1-s periodic Hamming windows, performed at a 1 kHz sample rate.

For the frequency response analysis, the stimulus and cone response were first detrended. *f*_*3dB*_ was calculated by a least squares linear fit between the last frequency that had a gain drop of less than 3 dB and the very next frequency to estimate the frequency at which the level of gain reached −3dB (*f*_*3dB*_). Filter slopes were determined by least squares linear fits from the peak frequency response (i.e., gain = 0 dB) to 20 Hz for the NTSCI or to 27.5 Hz for the SoS stimulus. Within these ranges, the frequency response was essentially linear for every cone analyzed, reflected by their high coefficient of determinations (NTSCI; r^2^ = 0.99 ± 0.003, 8 cones; SoS; r^2^ = 0.99 ± 0.002, 18 cones).

The ability of the frequency response to describe a cone’s response to the SoS stimuli was determined by the approach given in Rieke [12]. Here, the cone’s response to the stimulus was estimated by multiplying the light input and the interpolated frequency response in the frequency domain and converting the product back to the time domain by inverse Fourier transform.

The mean correlation of this predicted response with each individual measured response was then compared to the mean correlation of the cone’s mean response with each individual measured response. For example, using the values of a typical cone, when the average correlation between the predicted and individual responses was 0.94 and between the mean and individual response was 0.96, then the transfer function was said to predict 98% of the light-dependent structure of the cone’s measured response (S3A and S3B Fig). Using the noninterpolated frequency response reduced this value by 0.01%. The interpolated frequency response was therefore considered to be a reliable “full frequency” model of a cone’s response.

Impulse-response functions were generated by inverse Fourier transformation of the frequency response. Where SoS stimuli were used, the interpolated frequency response was used. To prevent the overall shape of average impulse-response functions being distorted by variations in response latency, the T2P of each individual cone impulse-response function was first time shifted to zero, the average and SEM calculated, and then the average impulse-response function time shifted back such that its T2P matched the group’s average T2P.

### Estimation of the time course of contrast adaptation

A description of the impulse-response functions associated with *f*_*3dB*_ values was developed using 24 impulse-response functions of L- and M-cones stimulated with the SoS contrast-switching stimulus. This description was used to generate simulated cone responses for which *f*_*3dB*_ values were known at all times. The *f*_*3dB*_ values were systematically varied at the different time points with 5-ms precision until a simulated cone response replicating both the magnitude and time course of the *f*_*3dB*_ change for cones was found. Full details are given in S4 Fig.

### Joint and conditional probabilities

Impulse response function T2P values were binned into 1-ms intervals. Contrast and luminosity values were calculated as described above using the “effective stimuli.” Contrast levels were binned into 0.0307 unit intervals ranging from 0.14 to 0.97. Luminosity levels were binned into 959 unit intervals ranging from 3,436 to 29,339.

The probabilistic relationships were generated via Bayes’ rule.

For joint probabilities:

$$p(r_{i},s_{i})=p(r_{i}\cap s_{i})$$ (2)

For conditional probabilities:

$$\begin{array}{c} p\left(r_{i}|s_{i}\right)=\frac{p(r_{i}\cap s_{i})}{p(s_{i})}\\ \text{or}\\ p\left(s_{i}|r_{i}\right)=\frac{p(s_{i}\cap r_{i})}{p(r_{i})}, \end{array}$$ (3)

where *p(r*_*i*_*)* is the probability that the T2P value occurs within bin *r*_*i*_, and *p(s*_*i*_*)* is the probability that a contrast (or luminosity) level is within bin *s*_*i*_.

### Statistics

All data are presented as mean ± SEM unless otherwise stated. Differences between groups were tested using two-tailed paired or independent *t*-tests as appropriate. Where the differences between means are given, the SEM was calculated as the Satterhwaite approximation of the standard error:

$$SE=\sqrt{\frac{s_{1}^{2}}{n_{1}}+\frac{s_{2}^{2}}{n_{2}}},$$ (4)

where *s* and *n* are the standard deviation and sample size.

## Supporting information

S1 Fig NTSCI—Non-normalized frequency responses and representative M-cone Joint and conditional probabilities. (PDF)

S2 Fig The SoS stimuli retained many of the characteristics of the natural stimulus. (PDF)

S3 Fig Cone responses to the SoS stimuli were well described by their frequency response relation. (PDF)

S4 Fig Estimation of the time course of adaptation. (PDF)

S5 Fig The normalized frequency response curves of the L-, M- and S- cone under voltage clamp conditions to high and low contrast stimuli using the SoS stimulus shown in Fig 2A. (PDF)

S6 Fig ‘*Effective*’ and ‘*stimulus*’ contrast and mean light intensity, and their distributions during various stimuli. (PDF)

S1 Table Light dependent structure predicted by the linear filter. (PDF)

S2 Table -3dB cut-off frequencies and frequency response curves slopes. (PDF)

S3 Table Properties of the impulse response functions. (PDF)

S1 Data Underlying numerical data and statistical analysis for 1A-E, 2A-B, 3A-D, 4A-E, 5A-Eii, 6A-Cii, S1 Table, S2 Table, S3 Table, S1A–S1C, S2, S3A–S3D, S4, S5, S6A and S6B Figs. (XLSX)

## Abbreviations

cGMP: cyclic guanosine monophosphate
FWHM: full width at half maximum
HCN: hyperpolarization-activated cyclic nucleotide-gated
I-V: current-voltage
L-cone: long-wavelength sensitive cone photoreceptor
LED: light-emitting diode
LGN: lateral geniculate nucleus
M-cone: mid-wavelength sensitive cone photoreceptor
NTSCI: natural time series of chromatic intensities
S-cone: short-wavelength sensitive cone photoreceptor
SEM: standard error of the mean
SoS: sum of sinusoids
SNR: signal-to-noise ratio
TEA: tetraethylammonium
T2P: time to peak
WN: white noise
