# Supplementary material for: A novel mechanism of cone photoreceptor adaptation
Source: PLoS Biol. 2017 Apr 12;15(4):e2001210. doi: 10.1371/journal.pbio.2001210 (PMC5389785; doi:10.1371/journal.pbio.2001210)
Supplement: S1 Fig — (PDF) [file pbio.2001210.s001.pdf]

S1 Figure: NTSCI - Non-normalized frequency responses and representative M-cone Joint and conditional probabilities.

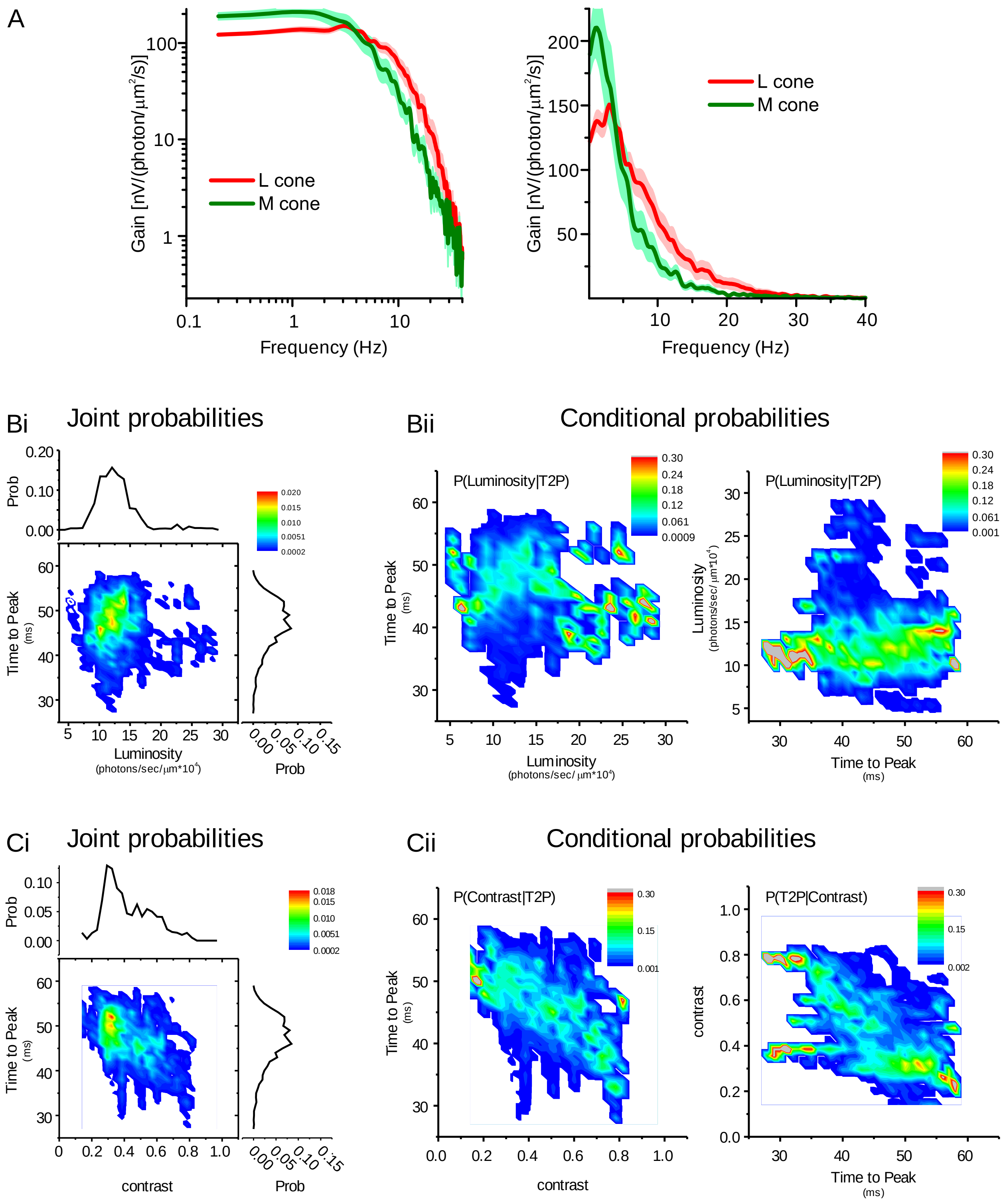

A) The frequency response curves for L- and M-cones for the entire NTSCI stimulus duration on log (left) and linear (right) scales. **Bi)** The joint (heat map) and marginal (line graphs) probabilities for the *'effective'* local mean light intensity (Luminosity) levels and the impulse response T2P for the representative M-cone shown in Fig 1E). The forward (**Bii**, left) and reverse (**Bii**, right) conditional probabilities for the same values shown in Bi). Overall B indicate a statistical independence between the two variables. **Ci**) The joint (heat map) and marginal probabilities (line graphs) for *'effective'* contrast levels and the impulse response T2P for the representative M-cone shown in Fig 1E). The forward (**Cii**, left) and reverse (**Ciii**, right) conditional probabilities for the same values shown in Ci). Overall C indicates a statistical dependency between the two variables. The data to generate this figure can be found in the S1 Data file.
