# Supplementary material for: A novel mechanism of cone photoreceptor adaptation
Source: PLoS Biol. 2017 Apr 12;15(4):e2001210. doi: 10.1371/journal.pbio.2001210 (PMC5389785; doi:10.1371/journal.pbio.2001210)
Supplement: S2 Fig — (PDF) [file pbio.2001210.s002.pdf]

S2 Figure: The SoS stimuli retained many of the characteristics of the natural stimulus.

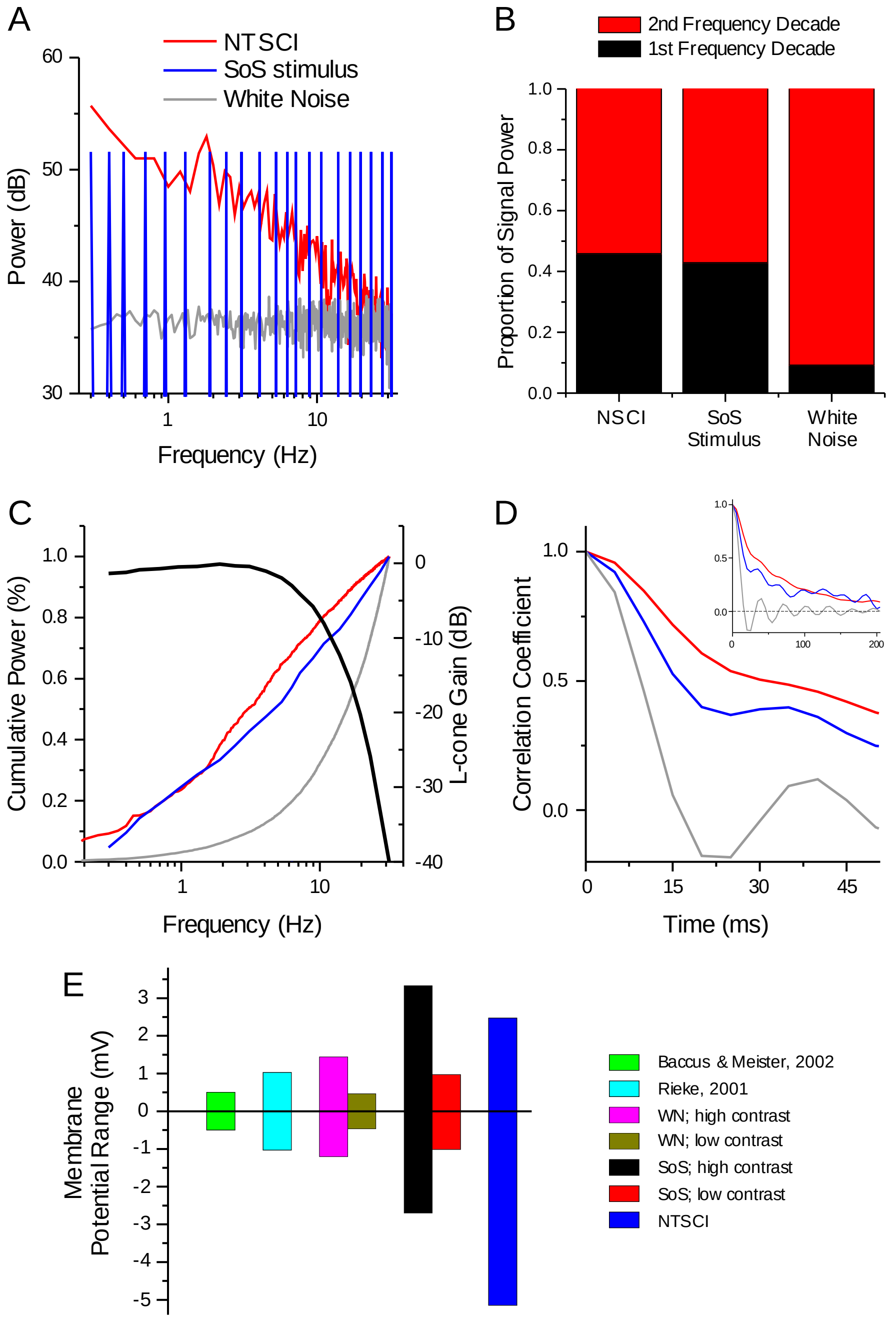

A) shows the power spectra of the NTSCI stimulus (red trace), a high contrast SoS stimulus (the sum of 21 sinusoids shown in Fig 3A, blue trace) and the average for 20 high contrast white noise stimuli (grey trace). The SoS and white noise stimuli are similar in that each frequency

used was of approximately equal power. However, as the frequencies used to construct the SoS stimulus were distributed almost equally along the logarithmic frequency axis, it retained several characteristics of the NTSCI. Like the NTSCI a large percentage of the spectral power occurs at the lower frequencies (**B** and **C**). For the NTSCI and the SoS stimulus the signal power was approximately equal in the first two frequency decades (**B**, starting at 0.3 Hz), a typical feature for a natural stimulus. By comparison, the white noise signal is dominated by frequencies within the 2nd frequency decade. The data shown have been normalized to the total power occurring within the two frequency decades. Similarly, for both the NTSCI and SoS stimuli the percentage of total stimulus power increases with frequency at a similar rate (**C**), and is quite distinct from that of white noise signals. Comparing these data with the frequency response amplitudes of L-cones (black) shows that most of the signal power for the NTSCI and SoS stimuli occurs within the physiological signalling range of the cones (roughly to -20 dB). For both stimuli, more that 50 % of the total signal power occurs in frequencies below f_{3dB}. For white noise signals, on the other hand, only 20 % of the total power occurs within this range. **D**) shows the autocorrelations of the stimuli, demonstrating that the serial correlation present in the NTSCI (red trace) and the SoS stimuli (blue trace) are highly similar. In both cases, the signals remain temporally correlated far longer than for white noise signals (grey trace). To make the comparisons in **A**) to **D**) fair, each noise signal had the same mean and *'stimulus'* contrast levels, and approximately the same total power, as the high contrast SoS stimulus. Additionally, the NTSCI and each noise signal were (sharp) low pass filtered at 31.7 Hz, the highest frequency used in generating the SoS stimuli. Note that filtering white noise signals adds a short-term serial correlation (15 ms to reach 0) with some minor rippling (**D**). White noise signals are

typically filtered at 30 Hz when used to stimulate lower vertebrate retinas. **E)** illustrates that the various stimuli differ in the effectiveness in modulating the cone's membrane potential. The cone response ranges found in our experiments with both the NTSCI and the SoS stimuli were much larger than we or previous studies have generated using Gaussian white noise stimuli. The NTSCI data is for the cone shown in Fig 1E-G, the SoS and white noise data are the averages for the 3 cones used in Fig 4B. The data to generate this figure can be found in the S1 Data file.
