# Supplementary material for: A novel mechanism of cone photoreceptor adaptation
Source: PLoS Biol. 2017 Apr 12;15(4):e2001210. doi: 10.1371/journal.pbio.2001210 (PMC5389785; doi:10.1371/journal.pbio.2001210)
Supplement: S3 Fig — (PDF) [file pbio.2001210.s003.pdf]

S3 Figure: **Cone responses to the SoS stimuli were well described by their frequency response relation**.

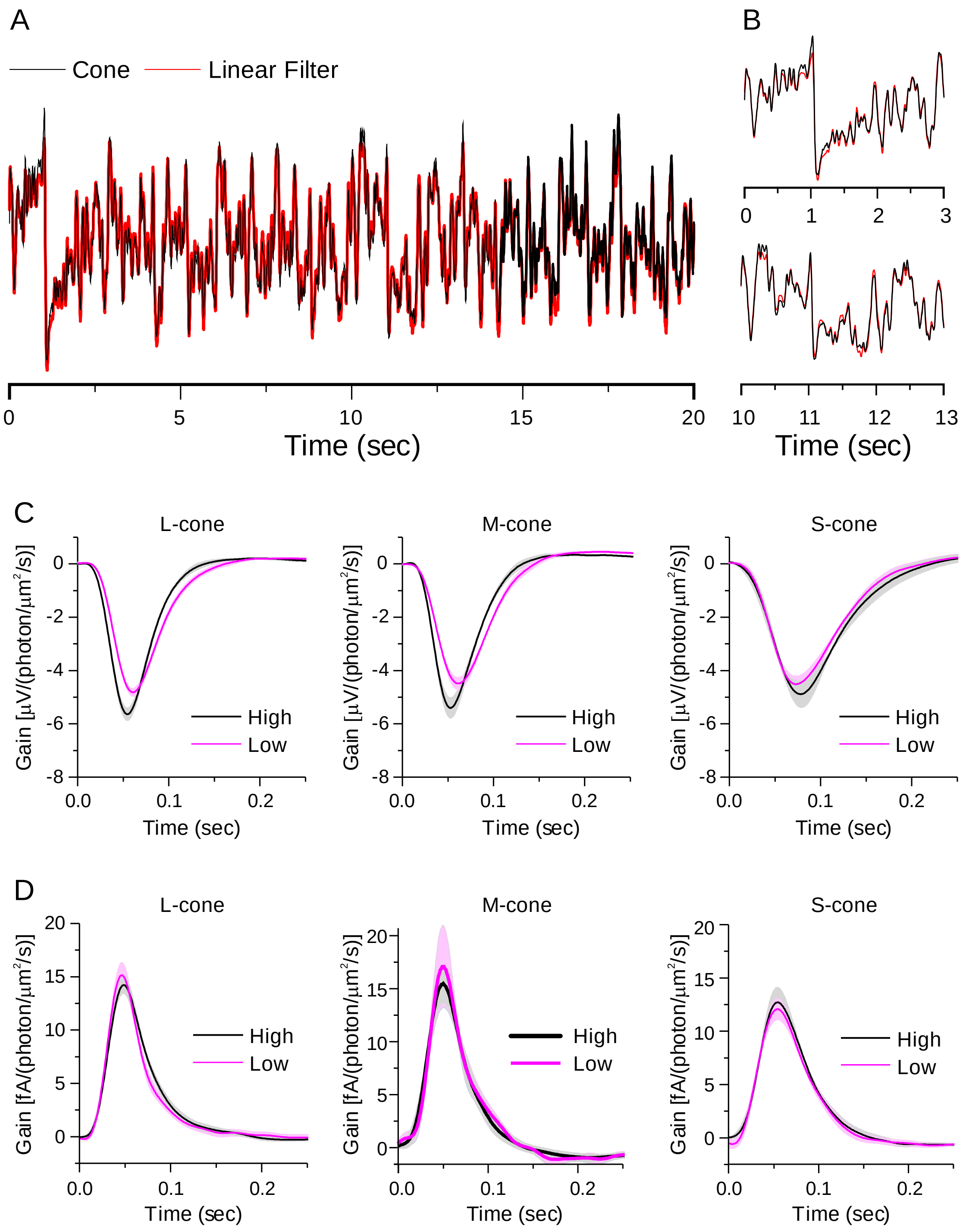

A) Comparison of a cone's mean response (black trace), and the response predicted by filtering the stimulus by the cone's frequency response relation (red trace) shows an almost complete overlap. This illustrates that the cone responses to the SoS stimulus were

essentially linear since stimulus-response characteristics were well described by their frequency response relation. This was quantified by determining the percentage of the light dependent structure predicted by the linear filter [1], which are shown in Table S1. In the example shown here of a M-cone stimulated with the high contrast version of the stimulus and measured in current clamp, the linear filter predicted 98.6 % of the light dependent structure. **B**) shows two sections of the cones mean response and the response predicted by the linear filter on an magnified time scale. See also Table S1. **C and D**) The voltage (**C**) and current (**D**) impulse response functions for L-, M- and S-cones for high and low contrast conditions when using the SoS stimulus shown in Fig 3A. The data to generate this figure can be found in the S1 Data file.

1. Rieke F. Temporal contrast adaptation in salamander bipolar cells. J Neurosci. 2001;21(23):9445-54.
