# Supplementary material for: A novel mechanism of cone photoreceptor adaptation
Source: PLoS Biol. 2017 Apr 12;15(4):e2001210. doi: 10.1371/journal.pbio.2001210 (PMC5389785; doi:10.1371/journal.pbio.2001210)
Supplement: S4 Fig — (PDF) [file pbio.2001210.s004.pdf]

S4 Figure: Estimation of the time course of adaptation

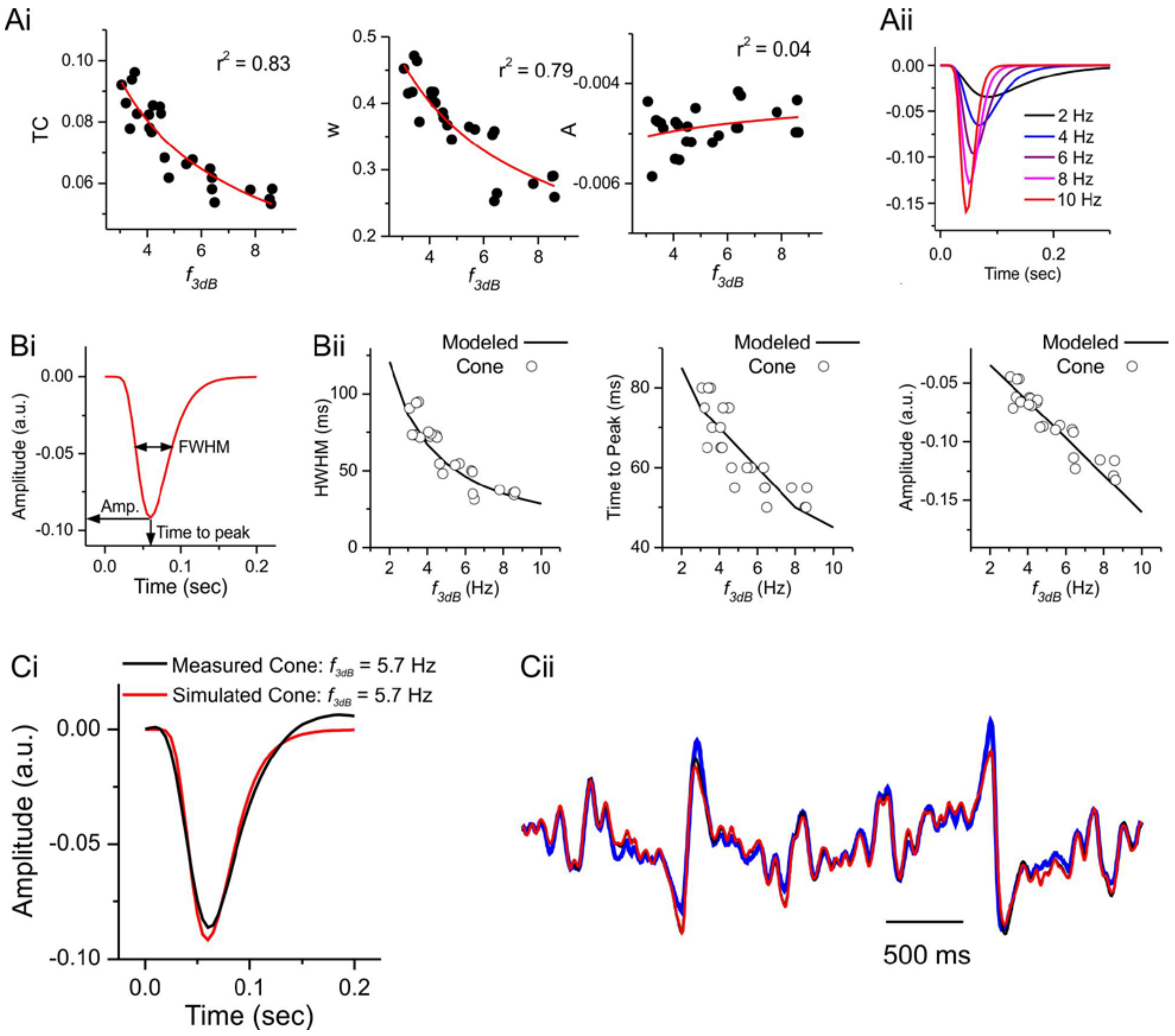

For the SoS contrast switching stimulus (Fig 3Ci), to accurately determine f_{3dB} at a given moment a time window of multiples of 4 seconds is needed (see Methods). This constraint meant that the time course of adaptation could not be determined by increasing the temporal resolution of the analysis simply by reducing the length of the time windows used. The time dependence of f_{3dB} shown in Fig 3D (upper panel) suggests that f_{3dB} starts changing soon after the stimulus contrast level switches from high to low and slowly declines over approximately the next 4 sec until it

reaches its final value. A similar pattern is seen when contrast levels switch back from low to high, although the transitional period is shorter. These results suggest the time course over which L- and M-cones adapt to changes in contrast is quite long. However, the 4 sec window periods needed to perform the analysis actually obscure the true behavior of the cones. For example, an abrupt switch from one f_{3dB} value to another will appear to develop over a 4 second period when using overlapping 4 sec window periods.

To better estimate the time course of the adaptational process, we simulated the change in f_{3dB} when measured with the 4 sec overlapping windows given a known time dependence of f_{3dB} using the following method. We estimated the relation between the shape of the voltage impulse response function of cones and f_{3dB}. Impulse functions were described by a lognormal function eq (1).

$$y = \frac{A}{\sqrt{2\pi w t}} e^{\frac{-\left(\ln \frac{t}{TC}\right)^2}{2w^2}} \qquad (1)$$

The mean (± SEM) r^2 of the lognormal function for the 24 cone impulse functions used was 0.99 ± 0.002, indicating that cone impulse functions are very well described by lognormal functions. Using data from L- and M-cones shown in Fig3D, we estimated the relation of the lognormal function parameters TC, w and A with f_{3dB} (Eq 2).

$$\begin{aligned} TC &= 0.167192 * f_{3dB}^{-0.529981} \\ w &= 0.805359 * f_{3dB}^{-0.502277} \\ A &= \frac{\sum A}{n} = -0.004871667 \end{aligned} \qquad (2)$$

With these relations (red lines **Ai)** we could calculate the impulse function of a cone for a given f_{3dB} (**Aii**). Next we tested how well these relations described the three most descriptive features of the impulse functions of cones: the amplitude (in arbitrary units: a.u.), the time to peak and the width at half maximal amplitude (FWHM) (**Bi**). As shown in **Bii** there is close agreement between the values obtained from cones, and from our calculated impulse response functions. Hence, our calculated impulse response function for a given f_{3dB} is a very good estimate of an impulse response function of a cone with the same f_{3dB}. In other words, we can accurately simulate the filtering characteristic of a cone that occurs at a given f_{3dB}. We show this more directly in **C**. In **Ci** the impulse response function of a cone with an f_{3dB} of 5.7 Hz (black line) and our simulated impulse response with the same f_{3dB} (red line) are in close agreement. **Cii** compared this cone's mean light response (blue line) and the response predicted by convolving its impulse response function with the stimulus in the frequency domain (black line). Also shown is the response predicted when the simulated impulse response function was convolved with the stimulus in the time domain (red line). To do this a 400 ms length of the stimulus was convolved in the time domain with the modeled impulse response, and the produce summed to generate a single time point for the simulated cone response. The procedure was repeated in advancing 5 ms steps (i.e. with 97.5 % overlap) to produce a simulated cone response for the full stimulus duration. In this way, the f_{3dB} of the simulated cone response was known at, and could be change for, each time point. As **Cii** shows all three responses strongly overlap indicating that our time domain convolutions using the simulated impulse response functions can predict the cone response as accurately as the frequency domain convolution using the cone impulse response function. Finally, we generated a time course by which f_{3dB} had to be

changed to account for the experimental results. For this we systematically varied the f_{3dB} at different locations until we found a simulated change in f_{3dB}, with results closely matching the measured change in f_{3dB}. This is shown in Fig 3D. The data to generate this figure can be found in the S1 Data file.
