# Supplementary material for: A novel mechanism of cone photoreceptor adaptation
Source: PLoS Biol. 2017 Apr 12;15(4):e2001210. doi: 10.1371/journal.pbio.2001210 (PMC5389785; doi:10.1371/journal.pbio.2001210)
Supplement: S5 Fig — (PDF) [file pbio.2001210.s005.pdf]

S5 Figure: The normalized frequency response curves of the L-, M- and S- cone under voltage clamp conditions to high and low contrast stimuli using the SoS stimulus shown in Fig 3A.

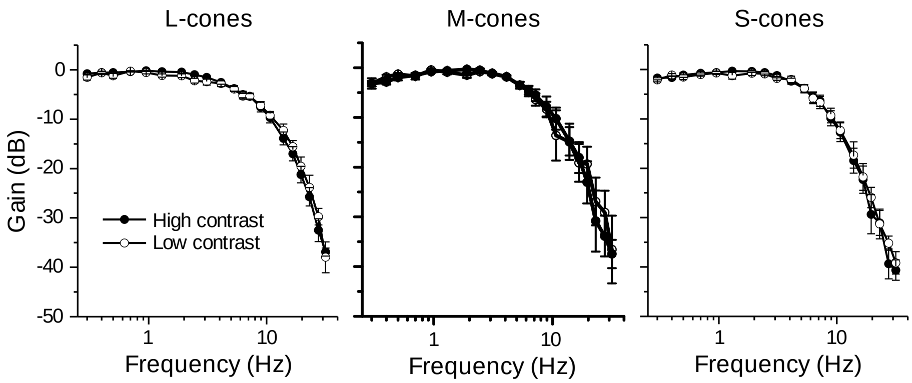

In this condition the frequency response was unaffected by stimulus contrast levels (also see S1 Table and S2 Table). Data shown as means ± SEM. The data to generate this figure can be found in the S1 Data file.
