# Supplementary material for: A novel mechanism of cone photoreceptor adaptation
Source: PLoS Biol. 2017 Apr 12;15(4):e2001210. doi: 10.1371/journal.pbio.2001210 (PMC5389785; doi:10.1371/journal.pbio.2001210)
Supplement: S6 Fig — (PDF) [file pbio.2001210.s006.pdf]

S6 Figure: *'Effective'* and *'stimulus'* contrast and mean light intensity, and their distributions during various stimuli.

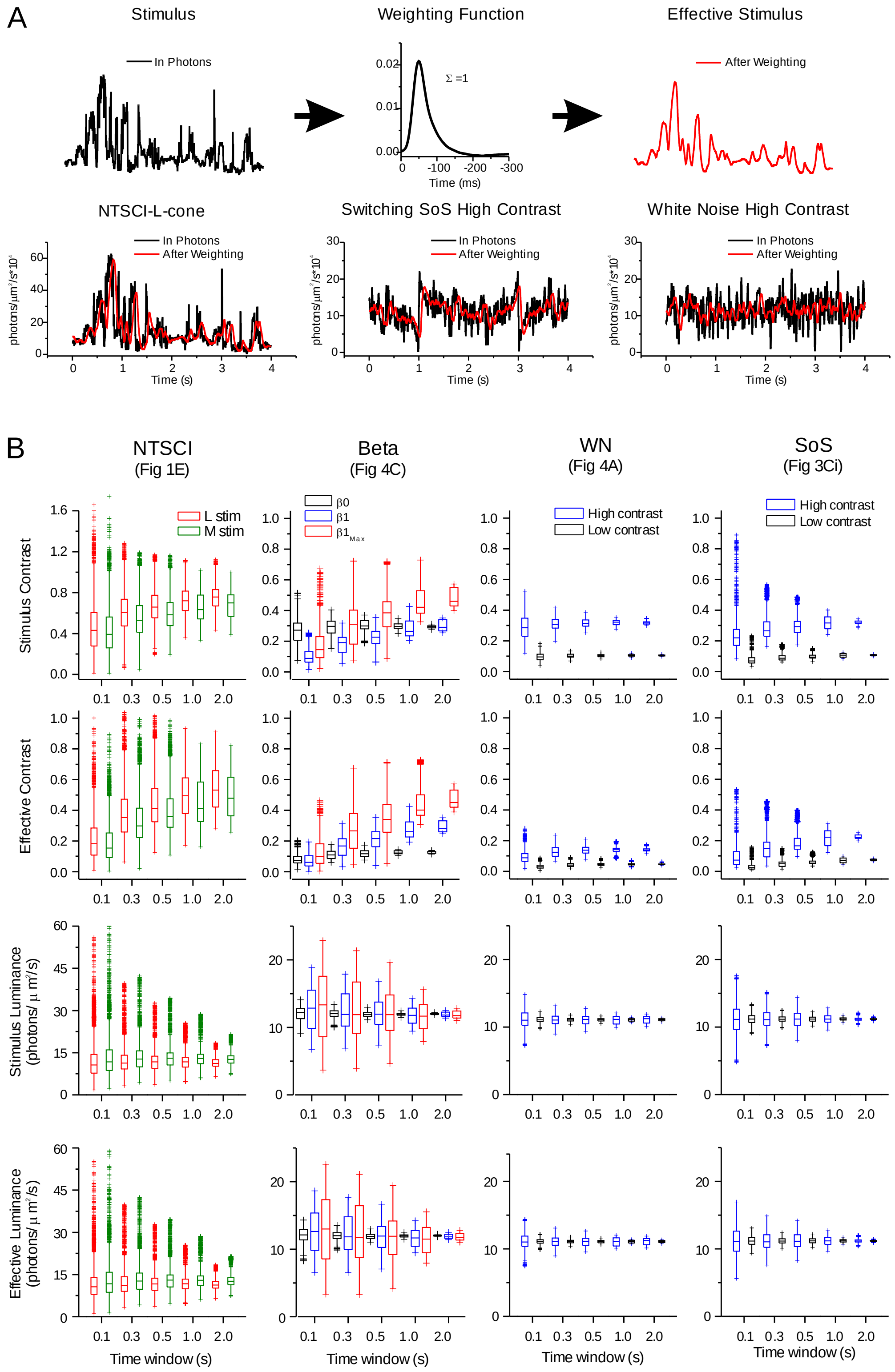

A) The cone's *'effective'* stimulus was calculated as follows. Cones have a relatively long temporal integration period (e.g. Fig 1C, 5B, 6A) so not all aspect of the stimuli will be equally available to the photoreceptors. For example, as fluctuations in stimulus intensity become faster they are increasingly encoded as a shift in the mean light level, and less as a flicker. Thus to estimate the *'effective'* stimuli available to cones, we weighted our various stimuli with a function derived from the temporal integration of the cone phototransduction at the mean light levels we used. To generate this function, we pooled all our L-, M- and S-cone results to both high and low contrast, when recorded in voltage clamp, and calculated the average. Its amplitude was then scaled such that all its elements summed to 1. Panel **A** demonstrates this procedure (upper) and shows representative 4 sec periods of several stimuli both before and after weighting (lower).

B) The distributions of *'effective'* and *'stimulus*' contrast and luminosity values when calculated over varying time periods. Within a stimulus time window of a given length its local mean intensity (luminance) is its mean value and its contrast is calculated as the SD of the values within the window divided by the mean value. The *'effective'* contrast and *'effective'* luminance were calculated this way using the *'effective'* stimulus and the *'stimulus'* contrast and *'stimulus'* luminance using the unweighted stimulus.

The level and range of contrast and luminance values calculated for a stimulus are contingent on the time interval over which it is calculated [1]. For instance, increasing the time interval over which the luminance and SD are calculated increases the average level of contrast and decreases the range of values generated for that stimulus. Without knowing the exact time periods etc. used by cones, it is not possible to calculate what contrast levels cones "perceived" under our stimulus conditions. For this reason, we avoided giving values where possible and instead talked in relative

terms, i.e. higher and lower. However, as we show in panel **B** when calculated over a wide range of time windows a broader range of contrasts, with higher median values and greater proportion of higher values, was delivered by our high contrast stimuli. For our artificial stimuli, a broader range of luminance values were also delivered by our high contrast conditions, however the median and mean values (see S1 Data file) for the high and low contrast stimuli were approximately the same. These distribution differences are qualitative the same for *'effective'* and *'stimulus'* contrast and luminosity.

Distributions are shown as boxplot, which indicates the 1st, 2nd and 3rd quartile, the whiskers show ± 1.5 the interquartile range and outliers are shown as (+). Each stimulus was randomly sampled, and at each location the local contrast and luminance level calculated using various time periods indicated on the X-axes. The number of random samples used were: NTSCI, 5000; WN and SoS, 2000 (1738 for 2sec periods); Beta, 1000 (684 for 2sec periods).

The data to generate this figure can be found in the S1 Data file.

1. van Hateren JH. Processing of natural time series of intensities by the visual system of the blowfly. Vision Res. 1997;37(23):3407-16.
