# Supplementary material for: A novel mechanism of cone photoreceptor adaptation
Source: PLoS Biol. 2017 Apr 12;15(4):e2001210. doi: 10.1371/journal.pbio.2001210 (PMC5389785; doi:10.1371/journal.pbio.2001210)
Supplement: S1 Table — (PDF) [file pbio.2001210.s007.pdf]

S1 Table: Light dependent structure predicted by the linear filter

	High contrast			Low contrast		
	% predicted	n cones	n stimulus repeats	% predicted	n cones	n stimulus repeats
L-cones						
Current Clamp	98.1 ± 0.56	6	7.3 ± 0.42	94.9 ± 1.92	6	7.0 ± 0.68
Voltage Clamp	97.0 ± 0.31	7	6.9 ± 0.40	90.8 ± 0.90	7	7.3 ± 0.71
M-cones						
Current Clamp	98.9 ± 0.21	6	6.3 ± 0.33	96.8 ± 1.07	6	5.7 ± 0.49
Voltage Clamp	95.9 ± 1.06	5	6.17 ± 0.65	89.5 ± 3.89	5	6.5 ± 0.99
S-cones						
Current Clamp	97.2 ± 0.36	6	6.2 ± 0.65	96.2 ± 0.85	6	6.8 ± 0.60
Voltage Clamp	97.1 ± 0.97	5	5.6 ± 0.81	92.0 ± 2.28	5	5.8 ± 0.58

Mean ± SEM percentage of the light dependent structure predicted by the linear filter [1] when using the SoS stimulus containing 21 sinusoids shown in Fig 3A. The data to generate this table can be found in the S1 Data file.

1. Rieke F. Temporal contrast adaptation in salamander bipolar cells. J Neurosci. 2001;21(23):9445-54.
