# Supplementary material for: A novel mechanism of cone photoreceptor adaptation
Source: PLoS Biol. 2017 Apr 12;15(4):e2001210. doi: 10.1371/journal.pbio.2001210 (PMC5389785; doi:10.1371/journal.pbio.2001210)
Supplement: S2 Table — (PDF) [file pbio.2001210.s008.pdf]

S2 Table: -3dB cut-off frequencies and frequency response curves slopes

	f_{3dB} *(Hz)* High contrast	f_{3dB} *(Hz)* Low contrast	n	p	Slope (dB/Hz) High contrast	Slope (dB/Hz) Low contrast	n	p
L-cone	6.0 ± 0.28	5.0 ± 0.34	6	0.003	-1.4 ± 0.05	-1.6 ± 0.07	6	0.014
M-cone	5.9 ± 0.44	5.0 ± 0.41	6	0.002	-1.4 ± 0.08	-1.7 ± 0.05	6	0.012
S-cone	3.5 ± 0.5	3.4 ± 0.28	6	0.79	-2.2 ± 0.12	-2.0 ± 0.09	6	0.27
L-and M-cones in 50 µM ZD7288	4.6 ± 0.75	4.6 ± 0.64	6	0.91	-2.0 ± 0.11	-2.1 ± 0.07	6	0.76

			f_{3dB}		Slope	
			High contrast	Low contrast	High contrast	Low contrast
L-cone	Vs	M-cone	p = 0.78	p = 0.98	p = 0.59	p = 0.14
S-cone	Vs	L-cone	p = 0.0015	p = 0.0054	p = 0.00018	p = 0.0038
S-cone	Vs	M-cone	p = 0.0059	p = 0.011	p = 0.00026	p = 0.016

-3dB cut-off frequencies (f_{3dB}) and frequency response curves slopes for all 3 cones types in high and low contrast conditions when recorded in current clamp (mean ± SEM) using the SoS stimulus shown in Fig 3A. Paired sample p values are given in the upper panel and independent sample p values given in the lower panel. The data to generate this table can be found in the S1 Data file.
