# Supplementary material for: A novel mechanism of cone photoreceptor adaptation
Source: PLoS Biol. 2017 Apr 12;15(4):e2001210. doi: 10.1371/journal.pbio.2001210 (PMC5389785; doi:10.1371/journal.pbio.2001210)
Supplement: S3 Table — (PDF) [file pbio.2001210.s009.pdf]

S3 Table: Properties of the impulse response functions

	Current Impulse Response			Voltage Impulse Response		
	High Contrast	Low contrast	p	High Contrast	Low contrast	p
L-cones	n = 7			n = 6		
Time to peak (ms)	48.4 ± 2.68	46.4 ± 2.30	0.43	54.0 ± 1.57	60.3 ± 2.04	0.009
FWHM (ms)	48.0 ± 4.07	43.8 ± 4.30	0.11	48.0 ± 1.19	54.8 ± 2.06	0.003
M-cones	n = 5			n = 6		
Time to peak (ms)	50.0 ± 5.89	49.8 ± 6.34	0.75	52.3 ± 2.30	62.2 ± 2.20	0.008
FWHM (ms)	51.2 ± 8.43	52.0 ± 8.82	0.78	51.9 ± 3.95	61.5 ± 2.79	0.012
S-cones	n = 5			n = 6		
Time to peak (ms)	54.6 ± 4.55	54.4 ± 4.03	0.86	76.2 ± 5.15	73.5 ± 4.84	0.66
FWHM (ms)	61 ± 6.95	60.4 ± 6.45	0.78	90.1 ± 10.05	83.9 ± 6.98	0.53

			Time to peak		FWHM	
Current Impulse Response			High contrast	Low contrast	High contrast	Low contrast
L-cone	Vs	M-cone	p = 0.82	p = 0.64	p = 0.74	p = 0.43
S-cone	Vs	L-cone	p = 0.28	p = 0.13	p = 0.15	p = 0.067
S-cone	Vs	M-cone	p = 0.55	p = 0.56	p = 0.40	p = 0.47
Voltage Impulse Response						
L-cone	Vs	M-cone	p = 0.57	p = 0.55	p = 0.37	p = 0.085
S-cone	Vs	L-cone	p = 0.0064	p = 0.042	p = 0.0083	p = 0.0075
S-cone	Vs	M-cone	p = 0.0040	p = 0.071	p = 0.011	p = 0.022

Voltage Impulse Responses under high contrast					
	n	Time to peak (ms)	p Vs ZD7288	FWHM (ms)	p Vs ZD7288
L-and M-cones in ZD7288	10	75.5 ± 4.50		75.6 ± 7.06	
Pooled L-and M-cones	12	53.2 ± 1.35	0.000051	49.9 ± 2.05	0.0012
S-cones	6	76.2 ± 5.15	0.93	90.1 ± 10.05	0.25

The impulse response functions time to peak and full width at half maximum (FWHM) for all three cone-types using the SoS stimulus shown in Fig 3A. The upper panel shows data for the current and voltage impulse responses for high and low contrast conditions. Corresponding paired sample p values are also shown. Independent sample p values comparing across these groups are given in the middle panel. The lower panel only gives data for voltage impulse response functions in the high contrast condition and corresponding independent sample p values. Data is shown for L-and M-cone in the presence of 50 µM ZD7288, and from the upper

panel the pooled result for L and M-cones and the data for S-cone. Data shown as mean ± SEM.

The data to generate this table can be found in the S1 Data file.
